# Efficient phase coding in hippocampal place cells

**DOI:** 10.1103/PhysRevResearch.2.033393

**Published:** 2020-09-11

**Authors:** Pavithraa Seenivasan, Rishikesh Narayanan

**Affiliations:** Cellular Neurophysiology Laboratory, Molecular Biophysics Unit, Indian Institute of Science, Bangalore 560012, India

## Abstract

Neural codes have been postulated to build efficient representations of the external world. The hippocampus, an encoding system, employs neuronal firing rates and spike phases to encode external space. Although the biophysical origin of such codes is at a single neuronal level, the role of neural components in efficient coding is not understood. The complexity of this problem lies in the dimensionality of the parametric space encompassing neural components, and is amplified by the enormous biological heterogeneity observed in each parameter. A central question that spans encoding systems therefore is how neurons arrive at efficient codes in the face of widespread biological heterogeneities. To answer this, we developed a conductance-based spiking model for phase precession, a phase code of external space exhibited by hippocampal place cells. Our model accounted for several experimental observations on place cell firing and electrophysiology: the emergence of phase precession from exact spike timings of conductance-based models with neuron-specific ion channels and receptors; biological heterogeneities in neural components and excitability; the emergence of subthreshold voltage ramp, increased firing rate, enhanced theta power within the place field; a signature reduction in extracellular theta frequency compared to its intracellular counterpart; and experience-dependent asymmetry in firing-rate profile. We formulated phase-coding efficiency, using Shannon's information theory, as an information maximization problem with spike phase as the response and external space within a single place field as the stimulus. We employed an unbiased stochastic search spanning an 11-dimensional neural space, involving thousands of iterations that accounted for the biophysical richness and neuron-to-neuron heterogeneities. We found a small subset of models that exhibited efficient spatial information transfer through the phase code, and investigated the distinguishing features of this subpopulation at the parametric and functional scales. At the parametric scale, which spans the molecular components that defined the neuron, several nonunique parametric combinations with weak pairwise correlations yielded models with similar high phase-coding efficiency. Importantly, placing additional constraints on these models in terms of matching other aspects of hippocampal neural responses did not hamper parametric degeneracy. We provide quantitative evidence demonstrating this parametric degeneracy to be a consequence of a many-to-one relationship between the different parameters and phase-coding efficiency. At the functional scale, involving the cellular-scale neural properties, our analyses revealed an important higher-order constraint that was exclusive to models exhibiting efficient phase coding. Specifically, we found a counterbalancing negative correlation between neuronal gain and the strength of external synaptic inputs as a critical functional constraint for the emergence of efficient phase coding. These observations implicate intrinsic neural properties as important contributors in effectuating such counterbalance, which can be achieved by recruiting nonunique parametric combinations. Finally, we show that a change in afferent statistics, manifesting as input asymmetry onto these neuronal models, induced an adaptive shift in the phase code that preserved its efficiency. Together, our analyses unveil parametric degeneracy as a mechanism to harness widespread neuron-to-neuron heterogeneity towards accomplishing stable and efficient encoding, provided specific higher-order functional constraints on the relationship of neural gain to external inputs are satisfied.

## I Introduction

Biological neural systems encode environmental stimuli in the process of eliciting behavioral responses. From the standpoint of efficient information encoding, these coded representations are required to maximize the information conveyed by the external stimulus. Within an information-theoretic framework, efficient encoding translates to the requirement that the distribution of stimulus attributes match the encoding strategy [1–12]. Efficient information transfer and associated strategies involving stimulus distributions have been effectively employed to assess biological systems from the sensory coding perspective [2–6,8,10–12], from a single neuron perspective [7,13–15] and in understanding biochemical signaling cascades [16–22].

In biological systems, such efficient representations emerge despite two critical constraints. The first involves the ubiquitous expression of biological heterogeneities, manifesting as pronounced neuron-to-neuron differences in response properties and underlying molecular components, even between adjacent neurons of the same subtype in the same brain region. These differences imply that the precision in encoding should account for this nonhomogeneous nature of neural parameters and responses. The second constraint corresponds to the limited ranges of the encoding space, placing a requirement that neurons and their circuits employ this range judiciously so as to enhance coding capacity. For instance, if neurons employed their firing rate as the code for a specific attribute of the environmental stimulus, there is a limit on the range of this firing rate based on the cellular and molecular constraints on the neuron. How do biological systems ensure maximal information encoding of environmental stimuli in the face of ubiquitous biological heterogeneity, while constrained by limited coding spaces?

**Fig. 1:**
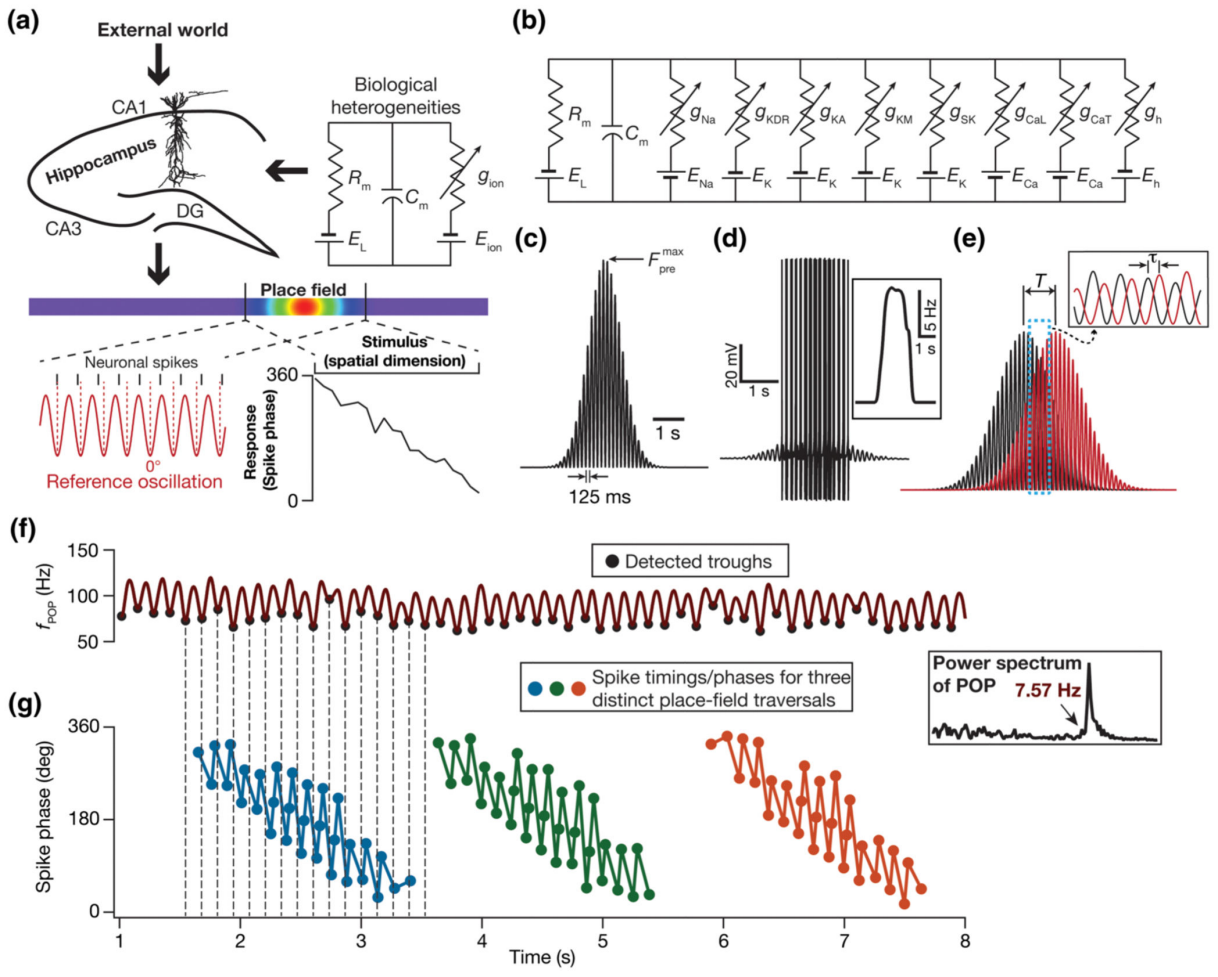
Developing a conductance-based neuronal model that incorporates biological heterogeneities to assess efficiency of the phase code. (a) Hippocampal rate and phase codes: A representation of hippocampal neurons, endowed with inherent biological heterogeneities in active and passive neuronal properties, receiving dynamic spatial stimuli from the external world. The rate code (violet-red along the rainbow spanning lower to higher firing) during an animal’s traversal along a one-dimensional track corresponds to a bell-shaped profile in the neuronal firing rate within the place field of the neuron. The concurrent phase code is derived from the phase of neuronal spikes with respect to an external reference oscillation. (b) Electric circuit equivalent of the conductance-based neuronal model employed in this study. (c), (d) A Gaussian modulated sinusoid (c) defined the probability distribution of activating 100 independent synaptic inputs arriving onto the conductance-based model during a place field traversal. In response to afferent synaptic activation, the neuronal model elicited a voltage response (d), with spike rate defining the rate code [(d), inset]. (e) Illustration of the temporal relationship between the probability distributions that govern the two adjacent place field afferent synaptic activation. *T* signifies the longer time scale that corresponds to the temporal distance (the travel time) between adjacent place field centers. *τ* characterizes the shorter theta time scale temporal difference between adjacent place fields, modeled as a phase shift in adjacent sinusoids at theta frequency (inset). For this illustration, *T* = 1000 ms; *τ* = 75 ms. (f), (g) 50 overlapping place field inputs were presented to the model and the cumulative firing rate (*f*_POP_) spanning all such presentations were computed (f). The oscillatory frequency of this cumulative firing rate was computed from its Fourier spectrum [(f), inset]. Phase coding emerges in the model as a precession of the phase of spikes elicited during each place field traversal (three traversals shown), computed with *f*_POP_ as the reference oscillation (g).

The hippocampus encodes space using rate and phase codes [23–27] through environment-specific firing in a dynamic subset of its population that are called place cells [Fig. 1(a)]. Whereas a rate code is defined by an increase in the firing rate of a neuron within its receptive field, the associated precession of neuronal spike phase relative to the extracellular rhythmic activity (~8 Hz; theta frequency) characterizes a phase code. The phase code, unlike the nonmonotonic rate code where the firing rate peaks around the place field center, manifests as a relatively monotonic dependence of the neuronal firing phase on the animal's spatial location [Fig. 1(a)] *within* a single place field [23–27]. Such monotonicity confers upon the phase code an enhanced potential for information transfer, with the ability to act as a fine-grained spatial code within a single place field. The motivation for this study on the phase code within a single place field [23–33] arises from the perspective of defining phase-coding efficiency and exploring the impact of biophysical heterogeneities on efficient phase coding.

In this study, we address questions on the cellular and molecular constraints behind how hippocampal neurons utilize the finite phase span (0 – 360°) available for efficiently encoding space during single place field traversals. In doing this, we first defined an efficient phase code as one that maximizes spatial information transfer. Specifically, we adapted Shannon’s entropy formulation and defined the maximization of mutual information between spatial *stimulus* and phase *response* to characterize high efficiency. This formulation allowed us to quantitatively assess information transfer efficiency of the phase code in hippocampal pyramidal neurons, in response to the afferent inputs that are dependent on the spatial location of the animal *within* a single place field.

Our questions involving cellular and molecular properties and inherent heterogeneities in these biological components demanded biophysically rich neural models that explicitly incorporate these components. While synaptic and intrinsic properties are predominant functional aspects at the cellular level, ion channels and their properties constitute the molecular level parameters. Therefore, we developed a conductance-based spiking model, designed specifically to match ion channel and intrinsic properties of hippocampal CA1 pyramidal neurons, for phase precession. This model was developed from a rate-based model for the emergence of phase precession [30], and differs in several aspects including the evaluation of exact spike timings, efficiency of the phase code, subthreshold voltage responses and experience dependence of place cell profiles and the incorporation of specific ion channels, intrinsic properties, and associated biological heterogeneities. We demonstrate that our model accounted for several signature electrophysiological characteristics of place cells within their respective place fields, including phase precession, the emergence of subthreshold voltage ramp, increased firing rate, enhanced theta power, and a signature reduction in extracellular theta frequency compared to its intracellular counterpart.

We employed our model for phase precession to then build a heterogeneous population of conductance-based models generated through an unbiased stochastic search. We defined the search space to span 11 neuronal parameters and assessed phase coding efficiency in thousands of models that sampled this space. At a molecular level, we found that the subspace of models that showed high-efficiency phase coding was scattered across the parametric space. Importantly, our results showed that the ion channel combinations required for achieving efficient phase coding neurons are nonunique. Specifically, a neuron could employ disparate parametric combinations of components at the molecular level to drive a well-defined synergistic balance between components at a cellular level to achieve efficient phase coding. We demonstrate that this parametric degeneracy [34,35] was intact even when we placed additional constraints on neural response properties and was a consequence of a many-to-one mapping between the parametric and functional spaces.

On the contrary, at a higher-order functional scale, we found an intricate counterbalance between neuronal gain (intrinsic excitability) and strength of external inputs (synaptic strength) to be a critical constraint for achieving phase-coding efficiency. Specifically, despite the vastness of a neuron’s parametric and measurement spaces, a confined negatively correlated regime in the synaptic-intrinsic plane characterized the subspace for efficient phase coding. These observations imply that the 11-dimensional parametric space collapses to a negative slope manifold on the intrinsic-synaptic plane for models that exhibit efficient phase coding, and that several nonunique parametric combinations could satisfy this tight functional constraint. Finally, through a change in the statistics of presynaptic input structure, we demonstrate that experience-dependent asymmetry in place field afferent inputs introduces predictive temporal shifts to rate and phase codes, with the change in the phase code constituting an adaptive shift to preserve efficiency.

In summary, our study builds a conductance-based model for phase precession that satisfies several key cellular signatures of place cells in hippocampal CA1 pyramidal neurons. Employing an information-theoretic approach, we derive a quantitative definition of efficiency in phase coding within a single place field. We show that phase-coding efficiency is tightly intercoupled to higher-order constraints involving a counterbalancing relationship between neural gain and the strength of synaptic inputs. Despite the expression of several biological heterogeneities and despite the requirement of such strong cellular-scale constraints, our analyses demonstrate that efficiency in phase coding could be achieved through disparate parametric combinations at the molecular scale. These conclusions unveil parametric degeneracy as a potent mechanism that enables several nonunique routes to achieve efficient coding while concomitantly maintaining homeostasis in neural excitability.

## II Defining Phase-Coding Efficiency

The fundamental objective of this study was to understand what constitutes the efficiency of phase coding in the functional space of a single neuron within its receptive field. Phase precession is a temporal encoding strategy exhibited by hippocampal neurons wherein the spike phases, within the neuron’s place field, progressively advance with respect to the extracellular activity. This results in a phase code, with phase being a monotonic function of the external space that is being encoded. Exploring the neuronal constraints that underlie the efficiency of spatial information transfer demonstrated by such a phase code demands a concise definition of encoding efficiency. To do this, we extended the concept of efficient coding from sensory systems [2–5,8] and single neurons [7] to phase coding within single place fields. Motivated by the efficient coding literature that recruits maximization of information transfer as a quantitative metric, we defined the efficiency of this phase code based on maximal mutual information between the *spatial stimulus* and *phase response*. This representation, which did not place any parametric constraints on the nature of phase precession, allowed for a generalized definition of encoding efficiency.

Mathematically, mutual information (MI) was defined as the difference between the response entropy and noise entropy [9]. With space and phase response constituting our variables, mutual information was given by

(1) I(ϕ;S)=H(ϕ)−H(ϕ∣S),

where *I*(*ϕ*; *S*) represented mutual information between stimulus (*S*; segregated into 20 distinct bins, where each bin constituted a different spatial stimulus) and neuronal phase response (*ϕ*) and *H*(*ϕ*|S) referred to the total noise entropy [9]. *H*(*ϕ*), the response entropy, was calculated as

(2) $$H(\phi )=-\sum_{j}\;p\left(\phi_{j}\right)\log_{2}p\left(\phi_{j}\right),$$

where *p*(*ϕ_j_*), the probability of occurrence of the *j*th phase bin (the 0–360° phase space was segregated into 360 bins; 0 ≼ *j* ≼ 359), was defined as

(3) $$p\left(\phi_{j}\right)=\sum_{i}\;p\left(\phi_{j}|s_{i}\right)p\left(s_{i}\right),$$

wherein the probability distribution of phases was derived by summing the conditional probability distributions of phases for various stimuli weighted by the probability of the stimulus. The total noise entropy was computed as

(4) $$H(\phi |S)=\sum_{i}\;p\left(s_{i}\right)H\left(\phi |s_{i}\right),$$

where *H*(*ϕ|S_i_*,) represented the conditional noise entropy for stimulus *S_i_*, and was computed as

(5) $$H\left(\phi |s_{i}\right)=-\sum_{j}\;p\left(\phi_{j}|s_{i}\right)\log_{2}p\left(\phi_{j}|s_{i}\right),$$

where *p*(*ϕ_j_*|s_i_) defined the conditional probability of the *j*th phase given the *i*th stimulus. Intuitively, response entropy captures the uncertainty in phase and noise entropy captures the uncertainty in phase despite the knowledge of the stimulus identity. Thus, noise entropy is that part of uncertainty that does not contribute any information about the stimulus and thereby is detrimental to information transfer. This explains mutual information as the difference between response and noise entropies [9].

More generally, the quantification proposed here to assess phase precession and the associated phase code alleviates problems associated with existing parametrized quantifications of phase precession (e.g., slope of the phase-space profile). In addition, the information-theoretic approach employed here provides a direct metric for the amount of spatial information (an environmental attribute) contained in the phase code (an attribute of neuronal firing) within a single place field.

### A Model description

Hippocampal pyramidal neurons exhibit significant neuron-to-neuron heterogeneities in terms of their intrinsic response properties and synaptic strengths, even *within the same subregion* of the hippocampus. The focus of our study is this subregion-specific (CA1) heterogeneity, which is distinct from the systematic location-dependent gradients in neuronal properties. A principal goal of this study is to understand the cellular and molecular constraints that are essential for highly efficient phase coding, especially in the presence of neuron-to-neuron heterogeneities. Finding these constraints demanded a neuronal model that was dictated by electrophysiologically measured properties at cellular and molecular scales, also including the statistics of neuron-to-neuron differences in these properties.

We constructed a single compartmental cylinder of 110-*μm* diameter (*d*) and 97-*μ*m length (*L*) of CA1 pyramidal neurons. The passive properties of the cylinder were specific membrane resistance, *R*_m_ = 40 kΩcm^2^ and specific membrane capacitance, *C*_m_ = 1 *μ*F/cm^2^. The geometric characteristics and the *R*_m_ were chosen such that the *passive* input resistance of the model [=*R*_m_ */*(*πdL*) = 119.3 MΩ] matched the electrophysiological values of ~120 MΩ, and the *passive* charging time constant (=*R*_m_ *C*_m_ = 40 ms) was ~40 ms [36,37]. The active properties included eight active ion channels (a total of nine channels, including the passive leak channel): fast Na^+^ (NaF), delayed rectifier K^+^ (KDR), *A*-type K^+^ (KA), *L*-type Ca^2+^ (CaL), calcium gated K^+^ (SK), hyperpolarization activated cyclic nucleotide gated (HCN), *M*-type K^+^ (KM) and *T*-type Ca^2+^ (CaT) channels. The kinetic schemes for these channels were derived from electrophysiological recordings from CA1 pyramidal neurons: fast Na^+^, CaL, KDR, and KA [38–40], HCN [41,42], CaT [43], SK [44,45], and KM [46], and the overall voltage dynamics evolved as [Fig. 1(b)]:

(6) $$C_{m}\frac{dV}{dt}=I_{\text{leak}}+I_{Na}+I_{CaL}+I_{CaT}+I_{HCN}+I_{KDR}+I_{KA}+I_{SK}+I_{KM}.$$

All channels except the SK channel were modeled using the Hodgkin-Huxley formulation, each involving one or more voltage (*V*) dependent differential equations to define the dynamics. SK channels were modeled using a six-state kinetic model [44,45]. Currents through the sodium channel, the HCN channel, and all potassium channels were modeled using the Ohmic formulation, but calcium channels were modeled using the Goldman-Hodgkin-Katz (GHK) formulation [47,48], to account for the large concentration gradient observed in the calcium ion. The model neuron was built within an 11-dimensional parametric space comprised of ten intrinsic parameters and synaptic strength. The maximal conductances associated with the individual ionic currents $(g_{\mathrm{leak}}=1/R_{m},\;{\bar{g}}_{\mathrm{Na}},\;{\bar{g}}_{\mathrm{CaL}},\;{\bar{g}}_{\mathrm{CaT}},\;{\bar{g}}_{\mathrm{HCN}},\;{\bar{g}}_{\mathrm{KDR}},\;{\bar{g}}_{\mathrm{KA}},\;{\bar{g}}_{\mathrm{SK}},\;{\bar{g}}_{\mathrm{KM}}),$ along with the decay time constant of calcium (τ_ca_; see below), were parameters that defined the intrinsic properties and *P*_max_ (the maximal synaptic permeability) represented synaptic strength. The reversal potentials for Na^+^, K^+^, and HCN channels were set at 55, –90, and –30 mV respectively.

The evolution of intracellular calcium as a function of calcium current (through voltage-gated calcium channels) and its buffering was modeled as in [49,50]

(7) $$\frac{d[\text{Ca}{]}_{i}}{dt}=-\frac{10000I_{\text{Ca}}}{36dptF}+\frac{[\text{Ca}{]}_{\infty }-[\text{Ca}{]}_{i}}{\tau_{\text{Ca}}}$$

where *F* is Faraday’s constant, *I*_Ca_ is the calcium current, the default value of the calcium decay time constant τ_Ca_ = 30 ms, *dpt* = 0.1 *μ*m represented the depth of the shell, and [Ca]_∞_ = 100 nM defines the steady-state value of cytosolic calcium concentration [Ca]*_i_*.

The current through the *α*-amino-3-hydroxy-5-methyl-4-isoxazolepropionic acid (AMPA) receptor (AMPAR) was modeled as the sum of currents carried by sodium and potassium ions [50]:

(8) $$I_{AMPA}(v,t)=I_{AMPA}^{Na}(v,t)+I_{AMPA}^{K}(v,t),$$

where

(9) $$I_{AMPA}^{Na}(v,t)=P_{\max}P_{Na}s(t)\frac{vF^{2}}{RT}\times \left(\frac{[\mathrm{Na}{]}_{i}-[\mathrm{Na}{]}_{o}\exp\left(-\frac{vF}{RT}\right)}{1-\exp\left(-\frac{vF}{RT}\right)}\right)$$

(10) $$I_{AMPA}^{K}(v,t)=P_{\text{max}}P_{K}s(t)\frac{vF^{2}}{RT}\times \left(\frac{[K{]}_{i}-[K{]}_{o}\exp\left(-\frac{vF}{RT}\right)}{1-\exp\left(-\frac{vF}{RT}\right)}\right),$$

where *P*_max_ represented the maximum permeability of the AMPA receptor. The relative permeability ratios *P*_Na_ and *P_K_* were equal and set to 1. *s*(*t*) was set as

(11) $$s(t)=a\left[\exp\left(-\frac{t}{\tau_{d}}\right)-\exp\left(-\frac{t}{\tau_{r}}\right)\right],$$

where *a* is a normalization constant, making sure that 0 ≼ *s*(*t*) ≼ 1, τ_r_, the parameter governing rise time, was set to 2 ms, and τ_d_, the decay time constant, was 10 ms [50].

All simulations were performed using the NEURON programming environment [51] at 34 °C with the simulation step size set at 25 *μ*s. Data analyses and graph plotting were performed using MATLAB and custom written software in the IGOR Pro environment.

### B Synaptically driven inputs and population activity of place cells

Place cell inputs to the model neuron were fed as probabilistic afferent activity impinging on 100 independently driven AMPAR synapses. Independent presynaptic trains of action potentials stochastically activated these synapses based on an overall firing rate pattern, modeled as a Gaussian-modulated cosinusoidal distribution, with the frequency of the sinusoid set at 8 Hz. This presynaptic activation profile of our conductance-based synapses was derived from the simplified rate model, where place-cell inputs were modeled as Gaussian-modulated cosinusoidal *currents* [30]. This modification was essential because a current-based input would not account for the driving-force dependence of synaptic currents or the kinetics of receptors [Eqs. (8)–(11)]. Therefore, the total afferent current to the model neuron arrives from *n* different place fields through multiple conductance-based synapses whose presynaptic firing rates were stochastically driven. Specifically, with reference to the *n*th place field within a linear arena, each synapse in a neuron received inputs with probability of occurrence at time *t* defined by [Fig. 1(c)]

(12) $$F_{\text{pre}}^{n}(t)=F_{\text{pre}}^{\text{max}}\left\{1+\cos\left[2\pi f_{0}(t-n\tau )\right]\right\}\exp\left(-\frac{(t-nT{)}^{2}}{2\sigma^{2}}\right),$$

where *f*_0_ represents the cosine wave frequency (8 Hz) that translates to the intracellular theta frequency, $F_{\mathrm{pre}}^{\max}$ regulates the maximal input firing rate, and *σ* defines the width of the Gaussian that controls the extent of the place field [30]. In this formulation [Fig. 1(e)], *T* signifies the longer time scale that corresponds to the temporal distance (the travel time) between adjacent place fields (modeled as a Gaussian) while *τ* characterizes a shorter theta time scale temporal difference between adjacent place fields (modeled as a phase shift in adjacent sinusoids at theta frequency). The standard deviation of the Gaussian distribution *σ* that governs the extent of single place fields was set as [30]

(13) $$\sigma =\frac{T}{2\sqrt{2}\tau f_{0}}.$$

This formulation [Eq. (12)] involving a single Gaussian defining the probability of presynaptic activation does not account for the different presynaptic neurons, each endowed with heterogeneous place field locations and differential synaptic weights in connecting the postsynaptic neuron [52–54]. However, the summation of the probabilities of the firing of each presynaptic neuron, weighted by their respective synaptic strengths (which mimics a Gaussian centered at the place field center of the postsynaptic neuron), would result in a probability distribution that is approximated by a Gaussian with appropriate scaling factor and standard deviation. Thus, the probability of presynaptic firing should be interpreted as that of a population of presynaptic neurons, each with differential synaptic strengths and heterogeneous place field locations, converging on a postsynaptic structure.

The interference pattern between inputs from nearby place fields results in a reduction in the frequency of the extracellular theta or the population firing rate [30]. To construct the population firing rate (*f*_POP_) within our conductance-based model framework, we presented inputs from 50 distinct place field locations to synapses of a given model neuron (defined with specific intrinsic properties and synaptic strength). Specifically, with reference to Eq. (12) representing a linear arena traversal, *n* ∈{1 … 50} reflects both a progressive shift in the center of the place field as well as a progressive phase shift in the theta time scale of individual place field inputs. The default values of *T* and *τ* were 180 and 10 ms, respectively.

For each value of *n* (∈{1 … 50}), the synapses in the model neuron were stimulated stochastically with the stimulation probability of each synapse sampled from the distribution in Eq. (12). The firing patterns of the model neuron to each of these 50 spatially distinct place field traversals were computed [Fig. 1(d)]. The spike times corresponding to each of these traversals were derived from these firing patterns, and converted to a binary time series (bin size 1 ms) indicating the presence or absence of a spike at a given time point. These binary time series were then summed across all 50 spatially distinct place field traversals to obtain the ensemble binary spike train that was then convolved with a Gaussian kernel to derive a smooth population firing rate profile [*f*_POP_; Fig. 1(f)]. The Fourier transform of this population activity was computed and the peak in the Fourier magnitude spectrum [Fig. 1(f), inset] was characterized as the population theta frequency (*f_θ_*), which represented the extracellular theta frequency [30].

### C Assignment of spike phases

In assigning neuronal spike phases with reference to the theta oscillation in the population firing rate (*f*_POP_), we first detected the troughs by determining the minima within each theta cycle [Fig. 1(f)]. These detected troughs of the population theta were all assigned a phase of 0°. We used these troughs to assign phase values to each spike corresponding to every place field input, with reference to the temporally aligned population theta oscillation (derived in the previous section). Specifically, let *t*_spike_ correspond to the timing of a spike in response to an arbitrary place field input. The population theta waveform was constructed from the ensemble output corresponding to all the 50 spatially distinct place field traversals, and encompasses the entire span of the linear arena [Fig. 1(f)]. The spike patterns corresponding to each of these 50 distinct place field traversals expectedly span a much-restricted spatial (and temporal) extent, implying that each spike would have a temporally aligned stretch of the population firing rate waveform [Figs. 1(f) and 1(g)]. Given this, for each *t*_spike_, we found two troughs of the population firing rate oscillation, one that immediately preceded *t*_spike_ (at time *t*_0_) another that immediately followed *t*_spike_ (at time *t*_1_). This implies that the neuronal spike (at *t*_spike_) occurred between two consecutive troughs (separated by a phase of 360°), at times *t*_0_ and *t*_1_, of the population firing waveform. Therefore the phase response *ϕ*_spike_ (in degrees) of the spike occurring at time *t*_spike_ with reference to *f*_POP_ was assigned as

(14) $$\phi_{\text{spike}}=360\frac{t_{\text{spike}}-t_{0}}{t_{1}-t_{0}}.$$

This assignment procedure was repeated for each spike elicited by the model neuron in response to all the 50 spatially distinct place field traversals with temporally aligned *f*_pop_. Given the cyclical nature of phase precession, these phases were warped (by shifting phase values by a constant number) such that the representation has actual phase values around 360° at spatial locations closer to the beginning of the place field.

### D Computing phase-coding efficiency of a model neuron

**Fig. 2:**
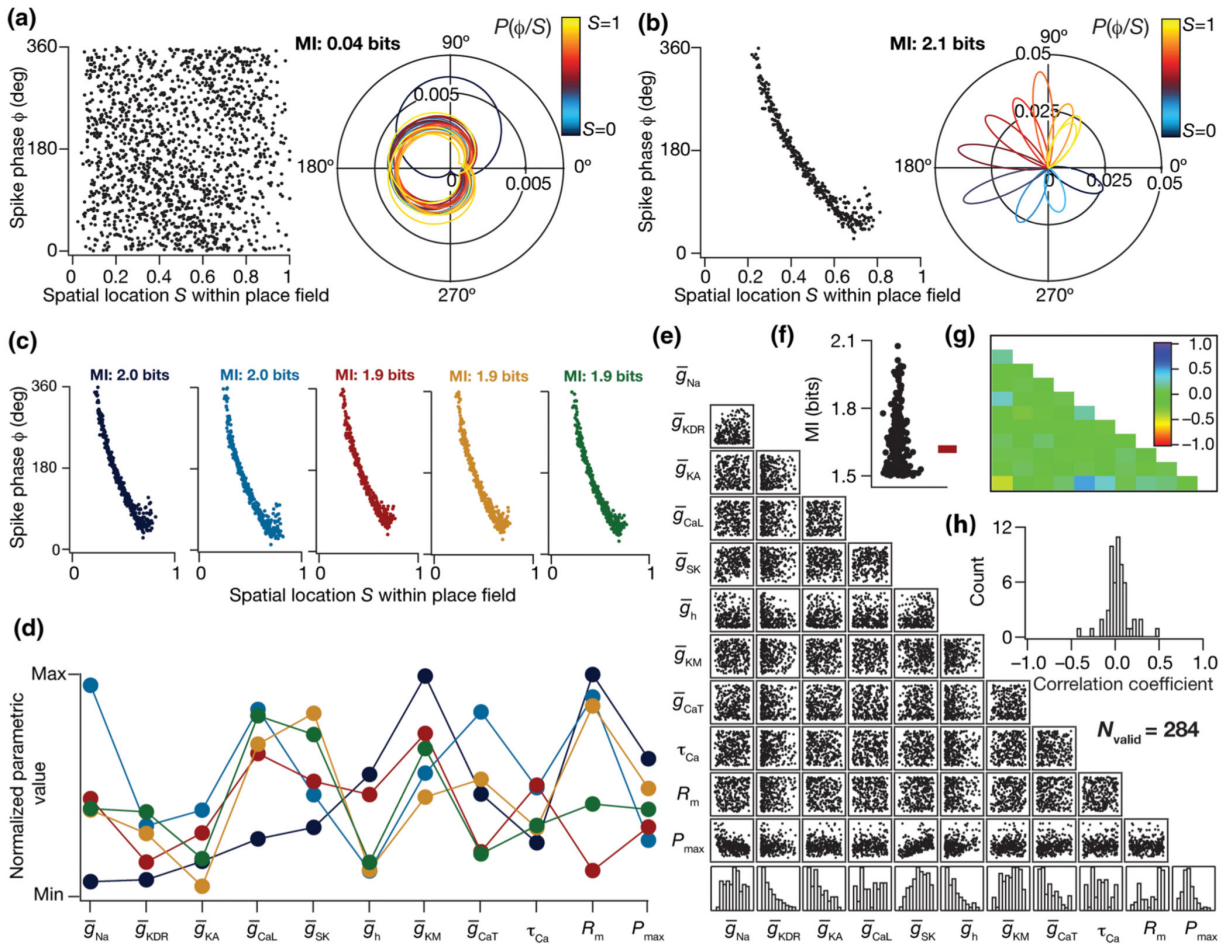
Degeneracy in efficient phase coding achieved through disparate combinations of neuronal components. (a), (b) Changes in neuronal intrinsic properties are sufficient to alter phase precession and the efficiency of the phase code. Phase profile of a model neuron that failed to express phase precession [(a), left] despite receiving identical synaptic inputs, thereby lowering the efficiency of information transfer (mutual information between firing phase and spatial location = 0.04 bits) through the phase code. A polar coordinate representation of conditional probabilities *P*(*ϕ*/*S*) of firing phases given the spatial bins within which the model received inputs [(a), right]. Theta phase precession [(b), left] and conditional probability distribution of phase responses [(b), right] for another model neuron (b) with a higher efficiency of information transfer (mutual information between firing phase and spatial location = 2.1 bits) through the phase code. The color code correspond to the different spatial stimuli (bins), and the normalized color code is provided along with the *P*(*ϕ*/*S*) graphs. (c) Phase precession of five example model neurons with very similar mutual information values, picked from a pool of 284 models classified as efficient phase coders due to an MI value greater than 1.5 bits. (d) Distribution of active and passive parameters that defined the five example models normalized between their respective ranges. (e) Scatter plot matrix of all 11 parameters that govern the 284 valid models, displaying pairwise correlations. The last row shows the histogram of the 11 parameters that defined these model neurons. (f) Beeswarm plot of the mutual information between firing phase *ϕ* and spatial location *S* for all the 284 valid models. (g) Pearson’s correlation coefficient quantifying the pairwise correlations, of scatter plots shown in (e). (h) Histogram of Pearson’s correlation coefficients of all 11 parameters, clustering around zero.

In assessing the efficiency of phase responses corresponding to the 50 spatially distinct place field traversals [Eq. (14)] obtained from our conductance-based model, we mapped the spike timings (and the corresponding spike phases) elicited for each place field traversal to a normalized place field space spanning 0 to 1. Specifically, with spike phase defined as the response and one-dimensional space constituting the stimulus, the construction of *p*(*ϕ_j_* |*s_i_*) [Eq. (5)] requires phase responses to multiple spatial traversals. In our formulation, the 50 spatially distinct place field inputs correspond to distinct spatial traversals, and the corresponding spike phase responses are those of the same model to stochastic presynaptic inputs [Eq. (12)] arriving onto the synapses from each of these 50 inputs. These 50 spatially distinct place field traversals are different only in terms of the place field center shifting by *T* ms for every consecutive place field input, along with a *τ* ms phase shift on the theta scale [Eq. (12)]. Therefore, to construct *p*(*ϕ*_j_ |s_j_), we superimposed the spike phase responses to multiple place field traversals by normalizing them with reference to their respective place field centers (Fig. 2).

Such normalization of spatial locations to 0–1 for each place field input, by accounting for their field center required an estimation of the spatial extent of each place field (so that no spike phases that belonged to the place field were omitted). The standard deviation (*σ*) of the place field Gaussian [Eq. (13)] offered an ideal metric for determining this, and we employed *2σ* on either side of the respective place field center as the extent of each place field. With the center and extent of each place field known, the space normalization of each spike phase was defined by mapping the left and right extremes of each place field to 0 and 1, respectively. Specifically, for the *n*th (0 ≼ *n* ≼ 49) place field traversal, the field center (*T*_cell_) and the left (*T*_L_) and right (*T*_R_) extremes were (see Supplemental Material Fig. S1 [55])

(15) $$T_{\text{cell}}=nT,$$

(16) $$T_{L}=T_{\mathrm{cell}}-2\sigma ,$$

(17) $$T_{R}=T_{\mathrm{cell}}+2\sigma .$$

Therefore, the normalized spatial location (0 ≼ *S* ≼ 1) for a spike occurring at *t*_spike_ for a specific traversal was given as

(18) $$S=\frac{t_{\text{spike}}-T_{L}}{T_{R}-T_{L}}$$

with *T*_L_ and *T*_R_ calculated for the specific traversal as in Eqs. (16) and (17). As all spike phases [Eq. (14)] corresponding to each of the place field traversals were now mapped onto a normalized spatial coordinate, all spike phases were superimposed to obtain a response (spike phase *ϕ*) vs stimulus (normalized space *S*) plot which was employed for computing *p*(*ϕ*|*S*) [e.g., Figs. 2(a) and 2(b)].

We computed *p*(*ϕ*|S) from this superimposed response-stimulus plot by binning the normalized stimulus axis *S* into 20 bins (see Supplemental Material Fig. S1 [55]), with each bin representing a spatial stimulus (*s*_i_, 0 ≼ *i* ≼ 19, in Eqs. (3)–(5). In computing the conditional probability distribution *p*(*ϕ*|s_i_) [Eqs. (3)–(5)], we pooled all the spike phases that belonged to the bin *s*_i_ [bin *i* spanned *i*/20 to (*i* + 1)/20 of the normalized stimulus axis *S*]. We computed the mean and variance of the phases within each stimulus bin *s*_i_ and constructed a normal distribution with these statistics to yield *p*(*ϕ*|*s_i_*) [e.g., Figs. 2(a) and 2(b)]. Finally, the mutual information between stimulus (*S*) and neuronal phase response (*ϕ*), *I*(*ϕ*; *S*), was computed employing the conditional distribution *p*(*ϕ*|*s*_i_) and the probability of occurrence of each stimulus bin *p*(*s_i_*), which was considered to be a uniform distribution (implying uniform traversal of space), using Eqs. (1)–(5).

In summary, the computation of *I*(*ϕ*; *S*) for a given conductance-based model neuron with a specified set of intrinsic properties [defined by parameters inherent to Eq. (6)] entailed the computation of the spike phase responses for each of the 50 spatially distinct place field traversals [Eqs. (12) and (14)], and computation of the response and noise entropies [Eqs. (2)–(5)] preceded by computation of *p*(*ϕ*|*S*) after normalization of the spatial coordinates [Eqs. (15)–(18)].

### E Model population replicating neuron-to-neuron differences in cellular and molecular properties

Functional measurements and ion channel properties of hippocampal neurons manifest neuron-to-neuron differences in the same subregion of the hippocampus. As a principal question addressed here was to understand the ability of neurons to act as efficient phase coders in the face of such neuron-to-neuron heterogeneity, our next step was to incorporate this in neuronal ion channel properties. We employed a multiparametric multiobjective stochastic search (MPMOSS) technique that involves assigning a broad search space for every parameter in the model, followed by construction of individual models through uniform random sampling of all the parameters for several thousands of times. Each model picked as a sample through such an unbiased stochastic search of a broad parametric space was evaluated for its ability to match with functional constraints to assess their validity [56–63].

We performed MPMOSS on 11 parameters that are critical to our model. The 11 model parameters that we included in the stochastic search were the nine maximal channel conductances $\left(g_{\mathrm{leak}}=1/R_{m},{\bar{g}}_{\text{Na}},{\bar{g}}_{\text{CaL},}{\bar{g}}_{\text{CaT},}{\bar{g}}_{\text{HCN},}{\bar{g}}_{\text{KDR},}{\bar{g}}_{\text{KA},}{\bar{g}}_{\text{SK},}{\bar{g}}_{\text{KM}}\right),$, the decay time constant of calcium (τ_Ca_), and the synaptic permeability (*P*_max_). The exhaustive search space for each of these parameters is given in Table I. Each of the 11 parameters in this multiparametric space (Table I) was uniformly sampled 11 000 times to generate as many unique models, and *I*(*ϕ*; *S*) was computed for each of these 11 000 conductance-based models (each with distinct intrinsic properties) employing the procedure outlined in the sections above.

In summary, we constructed 11 000 models that were heterogeneous across all parameters, with each model constituted by 100 stochastically activated synapses whose afferent inputs from 50 place fields were used to derive population activity.

## III Parametric Degeneracy in the Expression of Efficient Phase Coding

What constraints are essential on the 11-dimensional parametric space involving ion channel conductances to achieve efficient phase coding? To address this, we compared the phase coding efficiencies across all 11 000 models that received *identical* inputs during place field traversals, but were heterogeneous in terms of the cellular and molecular properties that defined them. Constructing and comparing the spike-phase profiles of such models provided us the first clue on the strong dependence of spike phase precession on neuronal properties. Two extreme examples elucidating this point are shown in Figs. 2(a) and 2(b), with the model in Fig. 2(a) eliciting bursts of spikes throughout the entire place field, resulting in a code where the phases assigned to individual spatial bins within the place field [Fig. 2(a), left] were not delineated [Fig. 2(a), right]. These overlaps in phase responses to spatial stimuli implied that this phase profile carried little information about the spatial location of the animal (MI = 0.04 bits).

Although both models received identical afferent inputs and had their phase codes computed with precisely the same procedure, only the model in Fig. 2(b) manifested a phase code that exhibited phase precession, with a clear monotonic relationship to spatial location. This translated to a broadly well-delineated range of phases being assigned to specific spatial bins [Fig. 2(b), right], implying that the spatial information contained in this phase code was higher [Fig. 2(b); MI = 2.1 bits] than the model in Fig. 2(a). The differences between these two models were limited to intrinsic properties, and not in the temporal structure of afferent network inputs or associated *T*/*τ* interactions [Fig. 1(e)] that define phase precession within our framework. Therefore, these observations pointed to a pivotal role for neuronal properties in the emergence of phase precession and in regulating the efficiency of the associated phase code.

What characteristics of the model parameters enable some of them to act as efficient phase encoders? To address this question, we assessed the cellular and molecular constraints that maximize the efficiency of a phase code by analyzing the properties of models that were highly efficient. We picked models that were endowed with a high value (>1.5 bits; Fig. S2 [55]) of mutual information *I*(*ϕ*; *S*) and considered them to be “MI-valid models“ or “efficient phase coders.“ Of the 11 000 models that were constructed, 284 models were classified as MI-valid models (~2.6%). The functional properties exhibited by this subset of 284 efficient encoders, both at the synaptic and intrinsic levels, would reflect the neural constraints that govern phase coding efficiency.

To understand the quantitative nature of this parametric subspace of efficient phase coding models, we first picked five (of the 284) models that were endowed with very similar phase precession profiles with high efficiency in spatial information transfer [Fig. 2(c)]. When we plotted the parameters associated with these five models [Fig. 2(d)], we found a lack of any clustering in the parametric combinations that resulted in these highly efficient models with very similar phase precession. This suggested the expression of degeneracy [34,35] in the manifestation of efficient phase codes, where disparate parametric combinations (representing distinct ion channels) yielded a similar function (phase-coding efficiency). To further validate our observations, we explored potential clustering in parameters by plotting the histogram for each of the 11 parameters associated with these 284 efficient models with MI ≥ 1.5 bits [Fig. 2(e), bottom row]. The broad distribution of parametric values that yielded these efficient models provided clear evidence for the expression of degeneracy in the emergence of efficient phase coding. To assess the presence of correlated expression of channel conductances underlying the efficient models, we plotted pairwise scatters of the 11 parameters from all the 284 models [Fig. 2(e)], and computed the associated correlation coefficients [Fig. 2(g)]. We found the pairwise correlations among model parameters to be weak, with the Pearson’s correlation coefficient spanning the range of –0.5 ≼ *R* ≼ 0.5 [Figs. 2(g) and 2(h)].

### A Ion-channel degeneracy in the concomitant emergence of efficient phase coding and neuronal excitability

The analyses thus far did not account for the characteristic intrinsic excitability of CA1 pyramidal neurons. While the MPMOSS picked out models based on their efficiency, there was no scrutiny on their electrophysiological equivalence to CA1 pyramidal neurons. We asked if imposing electrophysiological equivalence would alter the ability of disparate parametric combinations to yield efficient models that satisfy these constraints as well. We thus imposed a second layer of validation criteria on the 284 efficient models using the five measurements that were computed to characterize neuronal gain (intrinsic excitability)—resting membrane potential (RMP), standard deviation of RMP (σ_RMP_) to avoid fluctuations (consequent to channel interactions) in the emergence of a resting state, input resistance (*R*_in_), firing rate at 50 pA (*f*_50_), and firing rate at 250 pA (*f*_250_).

**Fig. 3:**
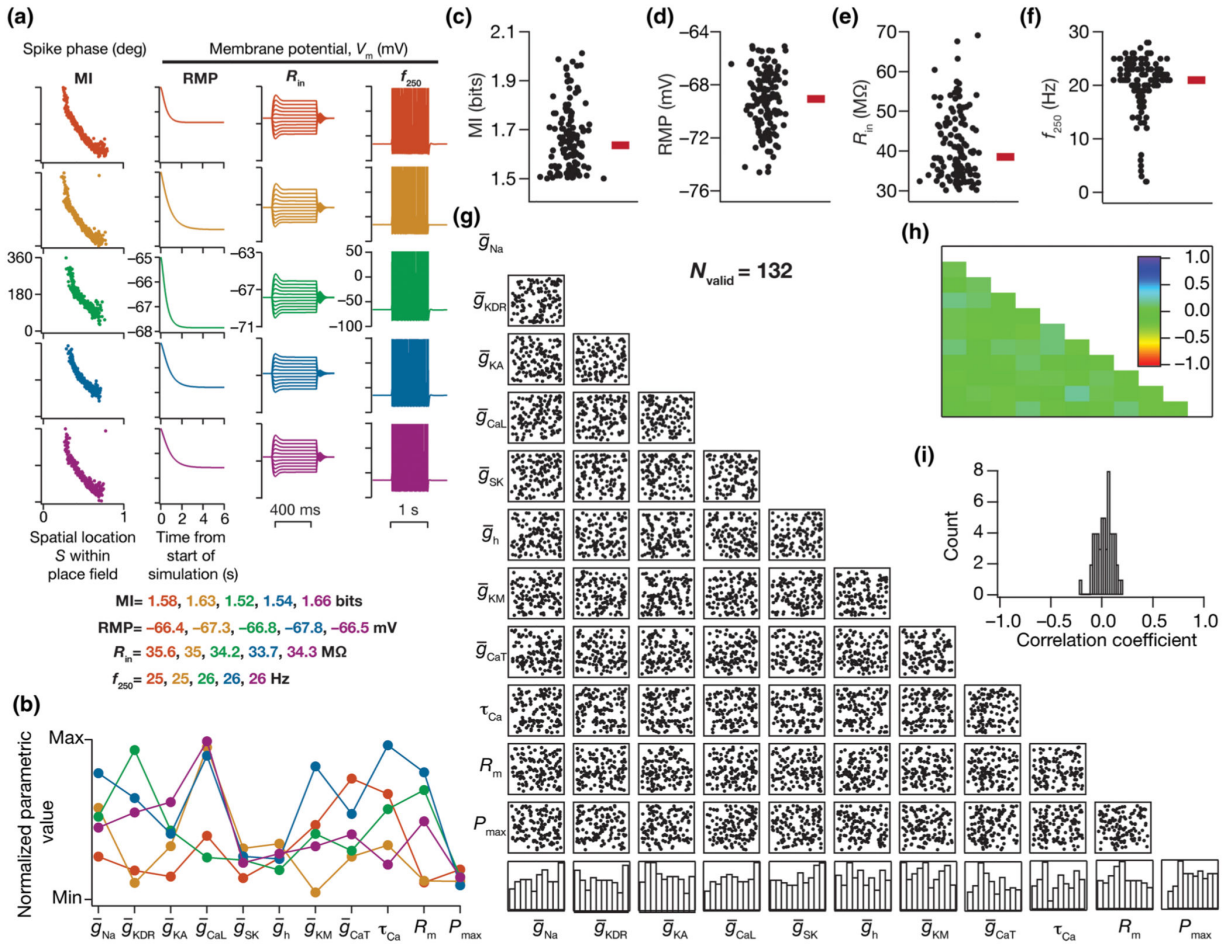
Degeneracy in efficient phase coding and concomitant physiological robustness achieved through disparate combinations of neuronal components. (a) The five color-coded rows represent different model neurons picked from a pool of 132 models that were classified as valid, based on high MI values and their intrinsic measurements satisfying electrophysiological bounds specified in Table II. The five models were chosen based on similarity in MI values as well as intrinsic measurements. The similarity of phase precession curves and mutual information (column 1), resting membrane potential (column 2), input resistance (column 3), and firing rate at 250 pA (column 4) across these five models may be noted. (b) Normalized parameter values that yielded the five models represented in (a), color-coded to represent the model identity. The parameter values are seen to span a large range (b), despite similarities spanning efficient encoding (MI) and intrinsic (RMP,*R*_in_, *f*_250_) measurements. The firing rate for 50-pA current injection, *f*_5_0, was identically zero and σ_RMP_ < 0.01mV for all 132 valid models (Table II). (c)–(f) Beeswarm plots of the mutual information between firing phase *ϕ* and spatial location *S* (c), resting membrane potential (d), input resistance (e), and firing rate for 250 pA (*f*_250_) current injection (f) for all the 132 valid models. (g) Scatter plot matrix showing pairwise correlations between parameters that underlie 132 models that were classified as valid based on MI values and excitability measurements. (h) The Pearson correlation coefficient matrix for all the pairwise correlations in the scatter plot matrix in (g). (i) Histogram of correlation coefficients shown in (h).

To measure RMP, the model neuron was allowed to achieve steady state (without injection of any current and without activation of any of the afferent synapses) for a period of 6 s [Fig. 3(a), second column]. The distribution of membrane potential values over the last 1 s (i.e., 5–6-s period) was employed to compute its mean and standard deviation, which were then defined as RMP and σ_RMP_ respectively. We injected the model neuron with pulse currents (each for 500 ms after steady-state RMP was achieved as above) of amplitudes ranging from –50 to 50 pA (with incremental steps of 10 pA each) and the resulting steady-state voltage response was recorded for each amplitude of current [Fig. 3(a), third column]. The steady-state voltage deflection from RMP was plotted as a function of injected current amplitude. The slope of the linear fit to this *V-I* plot was defined as the input resistance of the model neuron (*R*_in_). Finally, the firing rates at 50 and 250 pA were measured by injecting constant pulse currents of the respective magnitudes and computed as the number of action potentials elicited during a 1-s period. All these intrinsic measurements were made after allowing the RMP to stabilize for 6 s. For a model to be considered intrinsically valid, these measurements from the model ought to be within an experimentally valid range expressed by hippocampal CA1 neurons (Table II). This additional validation process resulted in 132 models that were efficient, and *concomitantly* satisfied multiple constraints on signature intrinsic excitability characteristics.

We assessed the parametric space underlying the 132 models that were efficient, and concomitantly satisfied multiple constraints on signature intrinsic excitability characteristics. To address ion channel degeneracy in this subpopulation, we first picked five example models that satisfied both sets of constraints. These models had similar or identical values not only for the mutual information measure [Fig. 3(a), first column] but also for each of the five intrinsic measurements [Fig. 3(a): columns 2–4; Table II]. Despite similarities in phase-coding efficiency and in the five intrinsic measurements, the underlying parametric values that defined these five models exhibited a broad distribution [Fig. 3(b)]. In assessing all the 132 valid models with high phase coding efficiency [Fig. 3(c)], we found intrinsic excitability measurements to exhibit heterogeneity [Figs. 3(d)-3(f)] within respective electrophysiological bounds (Table II). The parametric distributions of all 11 parameters defining these 132 models spanned a broad range rather than showing specific clusters [Fig. 3(g); last row histograms], with weak pairwise correlations across all parametric combinations [Figs. 3(g)-3(i)].

Together, these results unveiled ion channel degeneracy in the concomitant emergence of efficient phase coding and signature electrophysiological properties of hippocampal pyramidal neurons. As the 11-dimensional parametric space represents ion channel conductances, calcium decay, and receptor permeabilities, these constitute the *molecular* components that define our neuronal model. Our analyses of models exhibiting efficient phase coding clearly demonstrate the *absence* of strong constraints at the molecular scale, whereby disparate combinations of these parameters with weak pairwise correlations result in similar coding efficiency.

### B Ion channel degeneracy is mediated by differential and variable impact of individual ion channels on efficient phase coding

**Fig. 4:**
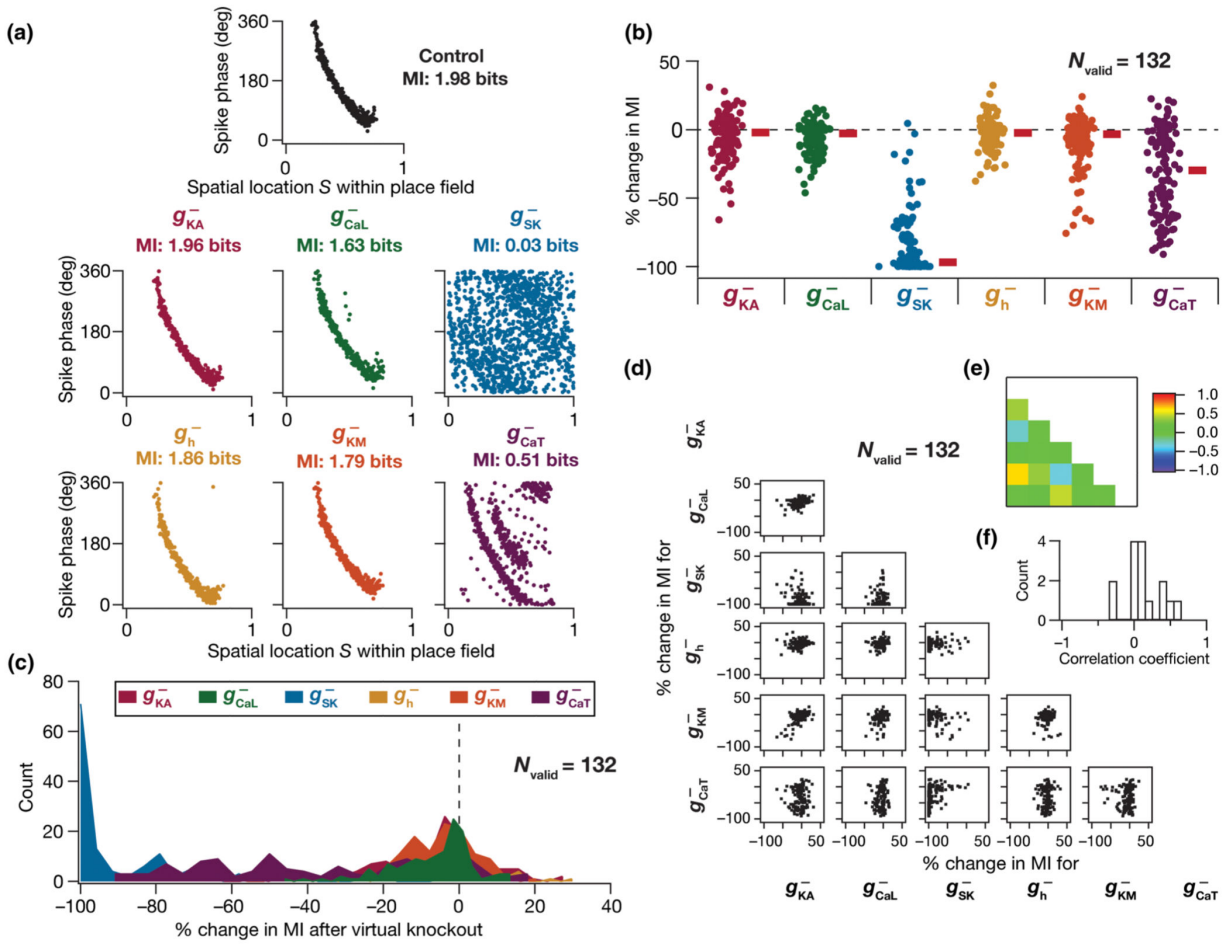
Virtual knockout models unveil differential and variable dependence of efficient phase coding on individual ion channels. (a) Phase precession profiles of an example model randomly picked from the 132 models that exhibited efficient phase coding and excitability homeostasis. Phase precession of this model with all the conductances intact (marked “Control“) and after virtual knockout of each of the six channels one at a time (the specific channel knocked out is mentioned on top of the respective phase-space plots). (b), (c) Beeswarm plots (b) and histograms (c) of percentage changes in mutual information values after virtual knockout of each channel from the population of 132 models. (d) Scatter plot matrix showing pairwise correlations between percentage changes in MI after virtual knockout of the six distinct channel subtypes in all 132 models. (e) The Pearson correlation coefficient matrix for all the pairwise correlations in the scatter plot matrix in (d). (f) Histogram of correlation coefficients shown in (e).

What aspect of the model mediated ion channel degeneracy? What roles do individual ion channels play in regulating efficient phase coding? To explore neural constraints on efficient phase coding at the molecular level, we assessed the relative contributions of ion channels to phase coding efficiency by using the virtual knockout strategy. Specifically, we virtually knocked out individual ion channels by setting the corresponding conductance to zero, with no changes to any other parameter or inputs to the selected model. We did not perform virtual knockout analyses on the NaF and KDR channels, because these virtual knockout models (VKMs) ceased spiking upon knocking out either of these channels, implying that spike phases could not be computed. Following knockout, we computed the phase precession and efficiency of each VKM and compared it with that of the same model when the channel conductance was intact and calculated the percentage change in mutual information. We repeated this procedure for all 132 valid models, and individually for the six active channel subtypes (Fig. 4; 132 × 6 = 792 VKMs, each subjected to 50 traversals).

The distribution of percentage changes in the mutual information in the population of models after virtual knockout of each of the six individual channels [Figs. 4(b) and 4(c)] unveiled the heterogeneity of the impact of these channels on efficient phase coding. Specifically, the impact of knocking out each of these channels was variable, whereby there was a strong effect on MI in some models with others showing no significant effect upon knockout of the same channel subtype. From the cellular perspective, the impact of knocking out different channels had differential effects on MI, with considerable cross-cellular heterogeneity in the relative contributions of individual channels to MI [Figs. 4(b) and 4(c)]. We asked if the impact of one channel subtype on efficient phase coding could predict the impact of another channel by comparing percentage changes in MI after individual channel knockouts in a pairwise manner [Fig. 4(d)]. Consistent with the correlation analyses on channel conductances [Figs. 3(g)–3(i)], we found that the pairwise correlations between percentage changes in MI after channel knockouts were very weak [Figs. 4(d)–4(f)]. The many-to-one relationship between different parameters (channel conductances) and coding efficiency, derived from variability in and weak correlations among the effects of individual channels, forms the substrate for ion channel degeneracy in the emergence of efficient phase codes (Figs. 2–4).

Although individual channels had differential and variable impact on phase coding efficiency, we noted that the phase code was heavily distorted upon knockout of SK channels and the *T*-type calcium channels in a large subset of models. These analyses offer a testable prediction on a critical role of SK and *T*-type calcium channels in determining the efficiency of the phase code. Together, our analyses unveil the expression of parametric degeneracy as a potential substrate for efficient coding in CA1 pyramidal neurons despite neuron-to-neuron heterogeneity in expression profiles. Ion-channel degeneracy ensures that efficient phase coding and intrinsic excitability characteristics are concomitantly maintained without the requirement of having individual ion channels at specific densities. These imply that different ion channel combinations contribute to similar functional outcomes, and manifest as variable and differential impacts of different ion channels on phase coding efficiency.

## IV Synergistic Balance Between Neural Gain and External Synaptic Inputs Defines Efficient Phase Coding

The analyses thus far demonstrated the absence of strong *molecular scale* constraints in the emergence of efficient phase coding, where disparate combinations of these molecular scale parameters elicited similar phase coding efficiency. Are there strong *cellular scale* constraints that are essential for the emergence of efficient phase coding models? What are the differences between models that showed efficient phase coding and those that did not, with specific reference to cellular-scale functional measurements? Is efficient phase coding simply a reflection of the firing rate of the model neuron during the place field traversal? Are there strong relationships between synaptic strength and intrinsic excitability in models that exhibited efficient phase coding? Are there correlations at the functional level between the efficiency of the phase code and the characteristics of the rate code (firing rate properties through the place field)?

**Fig. 5:**
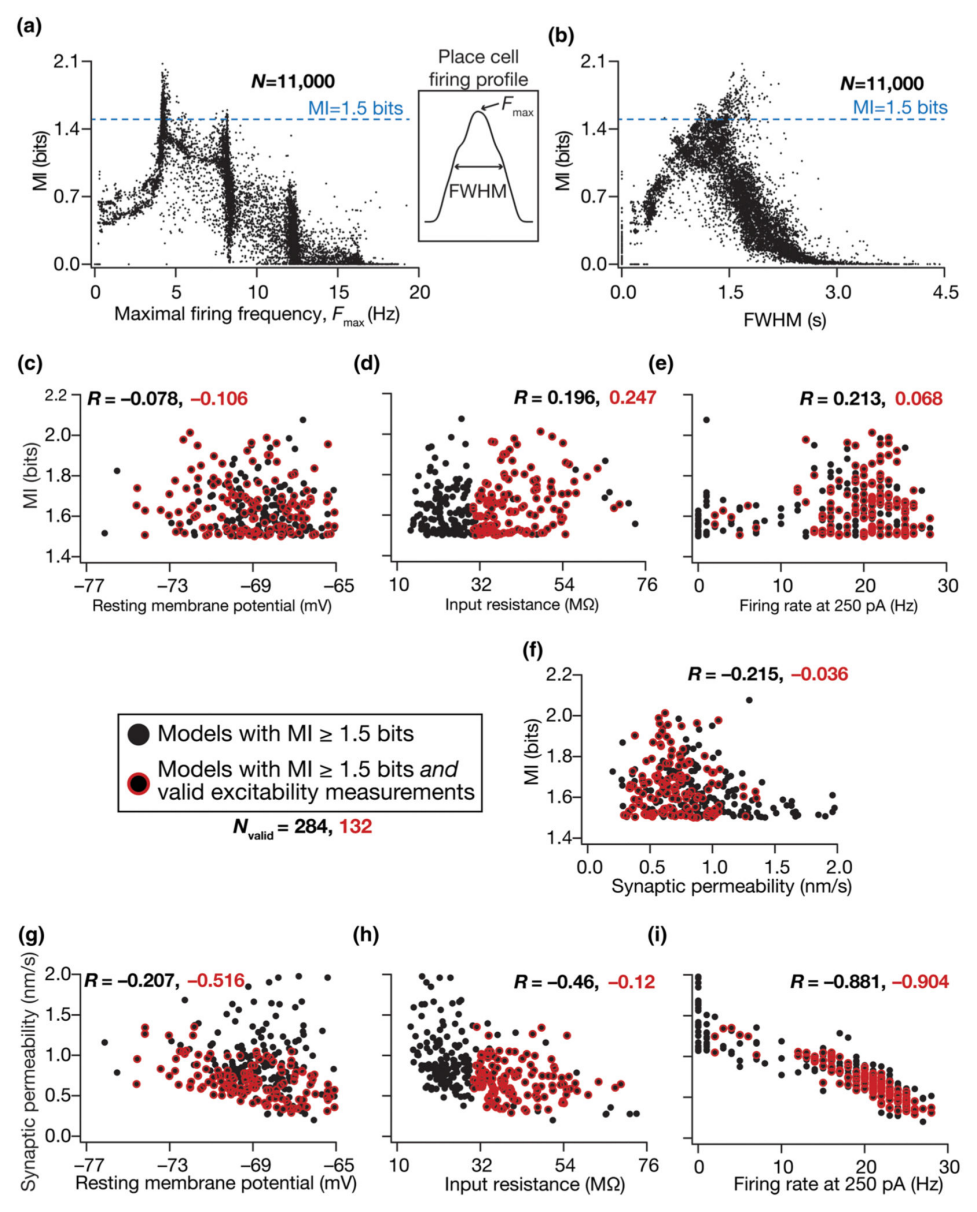
Synergistic functional interactions between synaptic strength and intrinsic excitability governed the emergence of efficient phase coding. (a), (b) Mutual information plotted as a function of the respective maximal firing rate (*F*_max_) and full width at half maximum (FWHM) of all 11 000 models. Inset between (a) and (b) shows a firing rate profile indicating *F*_max_ and FWHM of an example place-cell firing rate profile. (c)–(f) Scatter plots of mutual information vs resting membrane potential (c), input resistance (d), firing rate at 250 pA (e), and synaptic permeability (f) unveiled the absence of strong correlations between efficient coding and intrinsic/synaptic functional properties. (g)–(i) Scatter plots of synaptic permeability vs resting membrane potential (g), input resistance (h), and firing rate at 250 pA (i) revealed strong correlations between intrinsic firing frequency and synaptic strength (i). For all panels, black circles represented the population of models (*N*_valid_ = 284; from Fig. 2) that were endowed with an MI ≥ 1.5 bits, and a red outline around the black circles represented a subset of these models that were also endowed with valid intrinsic measurements (*N*_valid_ = 132; from Fig. 3). The values against *R* represent Pearson’s correlation coefficients for the respective color-coded scatter plots.

To address these questions, we analyzed the dependence of phase-coding efficiency (mutual information) independently on the properties of place field firing [*F*_max_, full width at half maximum (FWHM)], synaptic strength [*P*_max_ in Eqs. (9) and (10)], and each of the excitability measurements (RMP, *f*_250_, *R*_in_) for the 284 efficient phase coders and found very weak correlations (Fig. 5). We found that neurons with high phase coding MI were typically obtained when the peak firing rate was around 5 Hz [Fig. 5(a)] and when the FWHM was around 1 s [Fig. 5(b)], and there was no strong relationship between rate code properties and phase coding efficiency. We computed correlations between the phase-coding efficiency of the models (i.e., MI values) and each of the three excitability measurements, separately for the 284 efficient models and the 132 efficient and excitability-validated models [Figs. 5(c)–5(e)]. We found these correlations to be extremely weak [Figs. 5(c)–5(e)] thereby ruling out a direct well-defined relationship between intrinsic excitability and the model’s ability to efficiently encode space through phase. In addition, we found the correlation between model efficiency and synaptic strength for the two groups of valid models to be weak [Fig. 5(f)]. Together, these analyses ruled out strong relationships between phase coding efficiency and intrinsic excitability, synaptic strength, and place field firing properties.

Although there was no strong correlation at the functional level between the efficiency of the model and its intrinsic/synaptic properties, we found strong negative correlations between neuronal intrinsic properties and synaptic permeability for both valid model populations [Figs. 5(g)–5(i)]. This negative correlation was particularly strong between the neuronal firing rate and the synaptic permeability [Fig. 5(i)]. These observations suggest that neurons that occupy a negative slope manifold on the two-dimensional synaptic-intrinsic functional plane are capable of efficiently encoding spike phases. Importantly, these results show that efficient phase coding models can emerge *despite* heterogeneities in intrinsic properties [Figs. 3(d)–3(f) and 5(c)–5(e)] and in synaptic strength [Fig. 5(f)], as long as the synaptic drive counterbalances intrinsic excitability. Thus, the ability of intrinsic excitability and synaptic drive to *counterbalance* each other constitutes a critical cellular constraint in defining models with high-efficiency phase codes. Ion channel degeneracy described in the previous section demonstrates that this counterbalance could be achieved through distinct parametric combinations, thereby contributing to degeneracy in efficient phase coding. Although our search space for efficient models spanned an 11-dimensional parametric space, these analyses show that the search yielded models that occupied a negative-slope manifold on the synaptic-intrinsic functional space.

Thus far in our analyses, we first sorted models on the basis of their efficiency (Fig. 2) and among highly efficient models found a subset that was also endowed with signature excitability characteristics (Fig. 3). Instead, if models were initially sorted by whether they were endowed with signature excitability characteristics irrespective of what their MI values were, would these intrinsically valid models also be efficient phase coders? Would there be significant correlations between intrinsic properties and MI in this intrinsically valid model population? To address these, we identified models (Fig. S3A [55]; 1754 out of 11 000) that were intrinsically valid (Table II) irrespective of what their MI values were (Fig. 5). We found the MI values of these intrinsically valid models to span the 0–2-bit range (Fig. S3B), implying that they were not necessarily efficient and providing further evidence that efficiency in models was not a simple reflection of signature neuronal excitability properties. Additionally, we confirmed that our earlier (Fig. 5) conclusions on weak correlations between MI and intrinsic/synaptic properties extended to these intrinsically valid models as well (Figs. S3B-S3E). Importantly, we found that the synergy between synaptic and intrinsic properties, manifesting as high correlations between intrinsic measurements and synaptic permeability, was observed only in model populations that were endowed with high-efficiency phase codes [Figs. 5(g)–5(i)], but was notably absent in these models that were just intrinsically valid (Figs. S3F–S3H). These observations demonstrated that the synergy between intrinsic and synaptic properties was not a reflection of the stochastic search process, but manifested as an essential *constraint* in the emergence of efficient models and associated degeneracy.

Together, these results show that the tight counterbalance mediated by synergistic interactions between synaptic and intrinsic properties constitutes an important cellular constraint underlying the emergence of efficient phase coding. Our results also reveal degeneracy as a mechanism that recruits nonunique solutions from the parametric space (various parametric combinations that define model compositions) to achieve similar functional outputs (coding efficiency and neural excitability), mediated through tight constraints in the functional space (negative correlations in the synaptic-intrinsic plane, between neuronal gain and strength of external inputs). Importantly, while the structure of the synaptic inputs and synaptic weights plays a critical role in defining phase-coding efficiency, neuronal intrinsic properties need to be tightly coupled to these to maintain the *counterbalance* in achieving phase-coding efficiency.

## V Exploring Structure in the Parametric and the Measurement Spaces

The parametric solution subspace for models satisfying both constraints (models valid based on *only* efficiency and the ones valid based on *both* efficiency and intrinsic excitability) revealed significant expression of degeneracy. This was based on a considerable spread of all 11 parametric values across their respective entire ranges across valid models indicating that there was no noticeable clustering of these values that was reflective of efficiency or intrinsic validation. To further explore the prevalence of any hidden structure in the solution space, we applied dimensionality reduction techniques on the parametric and measurement spaces. Specifically, our dataset contains 11 000 models that contain three classes of models: (a) only intrinsically valid models (1622), (b) only efficient models (152), (c) both efficient and intrinsically valid models (132). Models that did not fire were excluded from these analyses, giving a total of 8579 model neurons for these analyses.

### A Parametric space

We performed linear (principal component analysis, PCA) and nonlinear (*t*-distributed stochastic neighbor embedding, *t*-SNE) dimensionality reduction techniques on the 11-dimensional parametric space (Table II) of the model population (Fig. S4 [55]). Specifically, we looked at the coefficients associated with the first three principal dimensions, with reference to the three classes of models mentioned above. We analyzed the distribution of class-specific coefficients in the reduced dimensional space obtained with PCA (Fig. S4A) or t-SNE (Fig. S4B), and found these distributions to be overlapping with each other. There were no clusters that distinguished between efficient models and models that were intrinsically valid. Together, these observations revealed the absence of any structure in the solution space that distinguished efficient models from others.

### B Measurement space

Motivated by our observations on the manifestation of strong correlation between synaptic strength and intrinsic excitability in highly efficient models (Fig. S5 [55]), we asked if there was structure in the measurements space that distinguished between efficient and intrinsically valid models. The measurement space is five-dimensional comprising resting membrane potential (RMP), input resistance (*R*_in_), firing rate at 250 pA (*f*_250_), the maximal synaptic permeability (*P*_max_), and the efficiency of a model’s phase code measured as mutual information between phase and space (MI). *P*_max_ was considered as a measurement dimension also in order to account for a synaptic measurement apart from intrinsic (RMP,*R*_in_, and *f*_250_) and efficiency (MI) measures. We performed PCA and *t*-SNE on the five-dimensional measurements space of the model population and plotted the class-specific coefficients along the first three principal dimensions (Fig. S5). We analyzed the class-specific distribution of these coefficients in the reduced dimensional space obtained with PCA (Fig. S5A) or *t*-SNE (Fig. S5B), and found these distributions to be overlapping with each other. There were no clusters that distinguished between efficient models and models that were intrinsically valid. These observations revealed the absence of any structure in the measurement space that distinguished efficient models from others.

Together, these analyses emphasized that complex nonlinear interactions among several parameters governed phase coding efficiency and intrinsic validity of the model population, thereby strengthening our conclusions on degeneracy in the parametric space. Specifially, these observations emphasize that several disparate combinations could yield similar functional outcomes *without* requirements of specific relationships between the parametric distributions [35,59,60,62,64]. In other words, changes in one parameter are not compensated for by changes in another specific parameter, but by changes in several other parameters and nonlinear interactions among them. These conclusions are also consistent with our prior conclusions from weak pairwise correlations between different parameters [Figs. 2(e)–2(h) and 3(g)–3(i)] and conclusions from our virtual knockout simulations showing differential dependence of phase-coding efficiency on individual ion channels (Fig. 4).

## VI Impact of Changes in Synaptic Input Structure on the Phase Code

**Fig. 6:**
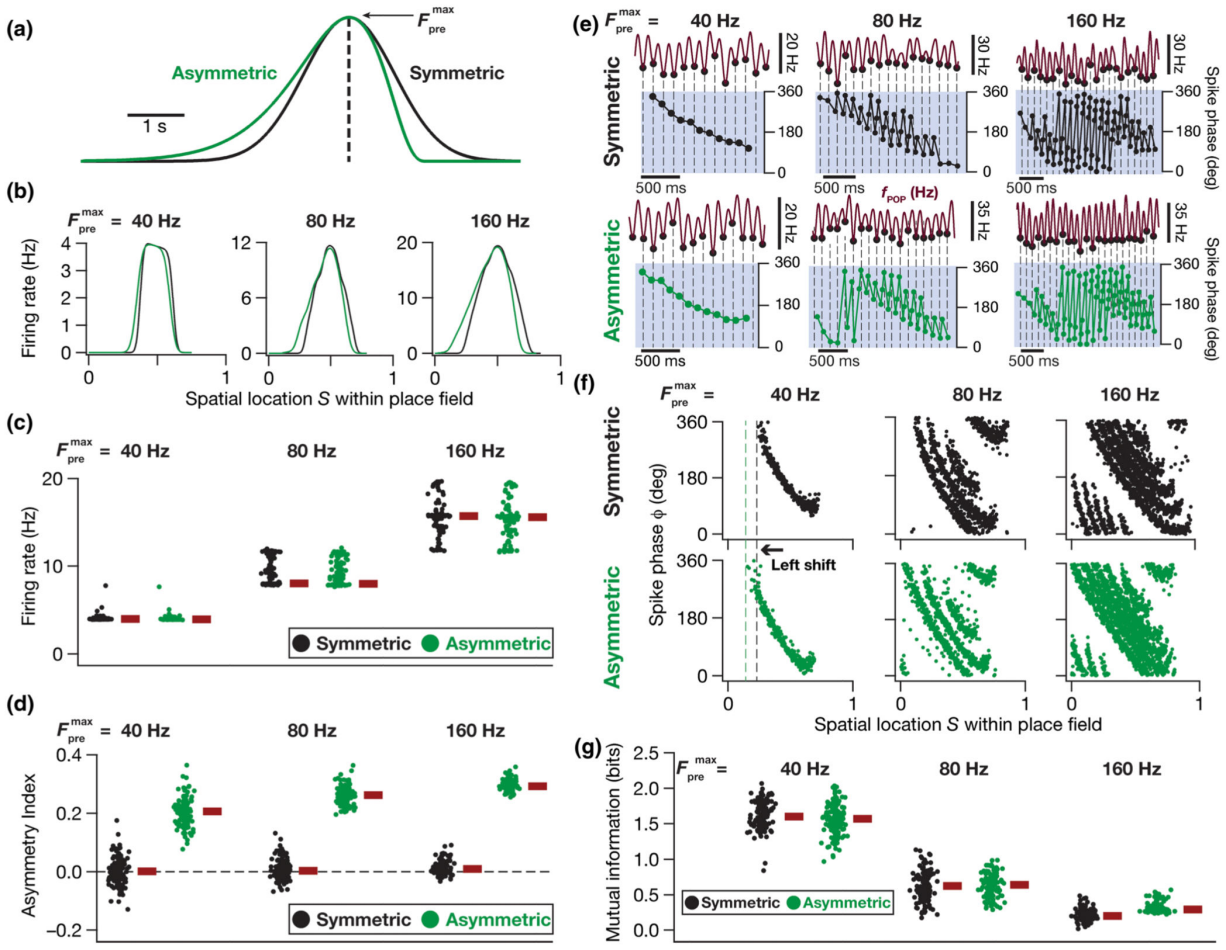
Experience dependence of rate and phase coding modeled through an asymmetric input distribution. (a) Symmetric (black) and asymmetric (green) profiles defining the probability distribution governing the activation of synaptic inputs arriving onto a model neuron during place field traversals. The symmetric profile is a theta-modulated Gaussian distribution while the asymmetric profile is a theta-modulated Erlang distribution. The peak $F_{\mathrm{pre}}^{\max}$ denotes the maximal presynaptic firing rate and the dashed black line indicates the center of the place field. (b) The three columns represent the firing rate profiles of an example model neuron that received symmetric (black) and asymmetric (green) inputs within its place field at three different presynaptic firing rates (40, 80, and 160 Hz). The place field extent is normalized between 0 and 1. (c), (d) Beeswarm plots of firing rates (c) and asymmetry indices (d) of all the 132 valid models receiving both symmetric (black) and asymmetric (green) input profiles at three different presynaptic firing rates (40, 80, 160 Hz). (e) Single-trial phase precession plots of the same model neuron as shown in (b) with reference to the theta oscillation. The three columns represent the model neuron receiving three different pre-synaptic firing rates (40, 80, and 160 Hz) and the two rows indicate symmetric (black) and asymmetric (green) cases of synaptic inputs. The black filled dots (in top traces) and dashed lines represent the troughs, which are aligned in time with the spikes of the model neuron (dots, bottom traces), in each panel. Note that the temporal scale bars for 500 ms become progressively shorter with increase in $F_{\mathrm{pre}}^{\max}$. (f) Multitrial phase precession plots of the same model neuron as shown in (b) and (e) for symmetric (black, top) and asymmetric (green, bottom) cases at three different presynaptic firing rates (40, 80, and 160 Hz) shown in the three columns. The leftward shift of the phase code for an asymmetric synaptic input profile may be noted for all three different presynaptic firing rates. (g) Beeswarm plots of mutual information between spike phase and spatial location within the place field, for all the 132 valid models, for both symmetric (black) and asymmetric (green) cases at three different presynaptic firing rates (40, 80, and 160 Hz).

There are theoretical and experimental lines of evidence for an experience-dependent asymmetric expansion of hippocampal place fields in the direction opposite the movement of the animal [31,33,65,66]. However, the place field model employed here involves a Gaussian that is symmetric with reference to the place cell center [Fig. 1(c)]. What is the impact of a change in the input structure such as an asymmetric afferent input activation on the place-cell rate and phase codes? How does such a change in the statistics of place field-driven afferent inputs onto a single neuron alter the efficiency of the phase code in the model? To address these questions, we replaced the symmetric Gaussian profile of afferent synaptic activation [Fig. 1(c)] by a horizontally reflected Erlang distribution to construct the asymmetric envelope while matching the area under the curve of these profiles [Fig. 6(a)]. With this formulation, each synapse in the model neuron received inputs whose probability of occurrence as a function of time was defined by an Erlang-modulated cosinusoidal distribution:

(19) $$F_{\mathrm{pre}}(t)=F_{\mathrm{pre}}^{\max}\left\{1+\cos\left[2\pi f_{0}(t-n\tau )\right]\right\}\times (nT-t{)}^{\alpha -1}\exp\left[-\beta (-nT-t)\right],$$

where all common parameters with Eq. (12) were identical in their description and function, and parameters *α*(=4) and *β*(=0.002) governed the extent of asymmetry. Similar to the theta modulation that we had introduced for the Gaussian, we incorporated theta modulation to the Erlang distribution by multiplying this function with an 8-Hz sinusoid. We maintained the same T-*τ* relationship as that of the Gaussian profile of synaptic activation [Figs. 1(e) and 2] for the 50 consecutive place field traversals, which now involve the Erlang distribution instead.

In comparing the implications of symmetric and asymmetric afferent activations on the place field rate and phase codes, we first constructed the firing rate profiles across each of the 50 different symmetric [Eq. (12)] or asymmetric [Eq. (19)] place field traversals for a given model neuron. To do this, the spike time responses of each model neuron to the 50 distinct symmetric or asymmetric place field inputs were convolved with a Gaussian kernel to produce instantaneous firing rate responses. These 50 firing rate profiles were finally averaged to produce the rate code of that particular model neuron [e.g., Fig. 6(b)], in response to either the symmetric or the asymmetric input profiles. To assess the sensitivity of the model to afferent input strength, we presented either symmetric or asymmetric inputs at three different maximal presynaptic firing rates ($F_{\mathrm{pre}}^{\max}$ = 40, 80, or 160 Hz). We computed an asymmetry index (AI) to assess the extent of asymmetry of the firing rate profile of a given model neuron:

(20) $$AI=\frac{A_{L}-A_{R}}{A_{L}+A_{R}},$$

where *A*_L_ denoted, for each model neuron, the area under the firing rate profile to the left of the place field center (0–0.5 on the normalized spatial axis) while *A*_R_ represented the same to the right of the place field center (0.5 to 1 on the normalized spatial axis). The firing rate profiles and their asymmetry indices were computed for each of the 132 concomitantly MI-and intrinsically valid models, at three different presynaptic firing rates, for both symmetric and asymmetric input profiles [Figs. 6(c) and 6(d)]. Predictably, the firing rate of the model cells increased with increase in $F_{\mathrm{pre}}^{\max}$ [Figs. 6(b) and 6(c)], irrespective of whether the activation profile was driven by a symmetric or an asymmetric distribution. As would be expected from the asymmetry in the afferent activation profile, we also found that the rate code displayed an asymmetry when the models were activated with the asymmetric distribution, with asymmetry progressively increasing with increase in $F_{\mathrm{pre}}^{\max}$ [Figs. 6(b) and 6(d)].

To understand the change in phase code as functions of asymmetry and $F_{\mathrm{pre}}^{\max}$ we computed the phase-space plots for each of these 132 models with each input configurations (symmetric vs asymmetric and three different values of $F_{\mathrm{pre}}^{\max}$) by computing the spike phases with reference to the respective *f_POP_*. We found phase precession to be overall similar for symmetric vs asymmetric synaptic activation profiles [Figs. 6(e) and 6(f)], which was confirmed by the similar efficiency in the phase code [Fig. 6(g)]. However, an important effect of asymmetry in the afferent activation profile was a leftward “predictive“ shift in the phase precession profile, which was consequent to the early intraplace field firing when neurons were activated with an asymmetric input profile and was observed for all values of $F_{\mathrm{pre}}^{\max}$ [Figs. 6(b) and 6(d)]. Specifically, although the models expressed similar efficiency values, their phase codes underwent a leftward shift to be able to preserve the high efficiency. In other words, models whose phase precession profiles looked very similar to their profiles in response to a symmetric activation curve (models that did not undergo a leftward shift) would not constitute the most efficient models in the asymmetric activation case.

These analyses revealed important differences in the phase code that emerged when the synaptic activation rate ($F_{\mathrm{pre}}^{\max}$) was changed [Figs. 6(e)–6(g)]. First, consistent with the rate code [Fig. 6(b)], the temporal spread of the extent of place field firing increased with $F_{\mathrm{pre}}^{\max}$ implying that the phase code now spread over a larger number of theta cycles [Fig. 6(e)]. Second, the increase in firing rate with increase in $F_{\mathrm{pre}}^{\max}$ implied that the cell spiked more than once during a single theta cycle [Figs. 6(e) and 6(f)], resulting in a reduction in the efficiency of the phase code with increase in $F_{\mathrm{pre}}^{\max}$, when all spikes were considered for the computation of mutual information [Fig. 6(g)]. Third, changes introduced to the phase code by increasing $F_{\mathrm{pre}}^{\max}$ were broadly invariant to whether the input activation profile was symmetric or not. Specifically, the increase in temporal extent of firing, the presence of multiple spikes within a single theta cycle, and the reduction in the efficiency of the phase code were all observed with increase in $F_{\mathrm{pre}}^{\max}$, irrespective of whether the synaptic activation profile was symmetric or asymmetric [Figs. 6(e)–6(g)]. Together, in our model, asymmetry in place field afferent inputs introduced predictive temporal shifts to the rate and phase codes, with the shift in the phase code constituting a stimulus-dependent adaptation of the code to the altered input statistics in order to achieve similar efficiency. These results imply that the phase code follows the stimulus statistics, which in this case is driven by the distribution of the afferent inputs as a function of space (as the traversal itself was considered uniform), thereby preserving the efficiency of information transfer.

### Theta modulation and symmetry profiles of subthreshold ramps in place-cell voltage responses

**Fig. 7:**
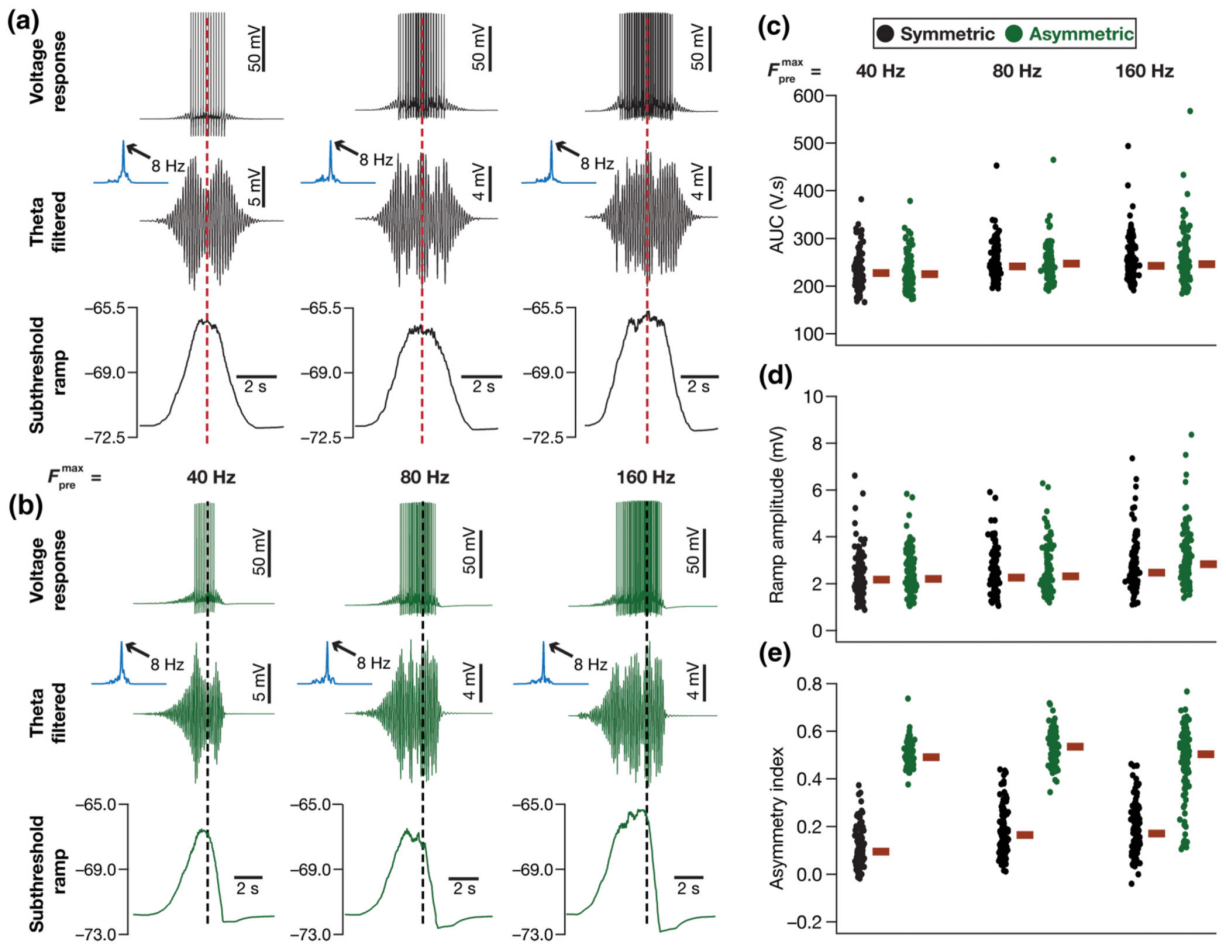
Model neurons exhibited enhanced theta power and subthreshold voltage ramp during place field traversals. For all panels in this figure, black and green correspond to symmetric and asymmetric synaptic activation profiles, respectively. (a), (b) The three columns for all plots represent three different values of the maximal firing rate of the presynaptic input $F_{\mathrm{pre}}^{\max}$ (40, 80, and 160 Hz from left to right). Top: Unfiltered voltage response of the model neuron for the symmetric (a) and asymmetric (b) synaptic activation during a place field traversal. Middle: The band-pass (4–10 Hz, theta band) filtered voltage response, represented in time domain along with its Fourier spectrum (blue) presented as an inset for symmetric (a) and asymmetric (b) cases. The area under the curve (AUC) for the blue Fourier spectrum traces in (a) were computed to be 234, 281, and 303 V s, and for those in (b) were computed to be 253, 290, and 287 V s, respectively for $F_{\mathrm{pre}}^{\max}$ = 40, 80 160 Hz. Bottom: The subthreshold voltage response profile was obtained by median filtering the voltage response, for the symmetric (a) and asymmetric (b) cases. The dashed red lines in panel (a) and the dashed black lines in panel (b) represent the respective centers of the place fields. The traces shown in (a) and (b) are from the same model cell employed in Fig. 6(b). (c)–(e) Beeswarm plots of AUC of the Fourier spectrum of the band-pass filtered signal (c), the subthreshold ramp amplitude (d), and asymmetry index of the subthreshold ramp (e) for all the 132 models. Measurements corresponding to symmetric (black) and asymmetric (green) synaptic activation profiles for the three values of $F_{\mathrm{pre}}^{\max}$ (40, 80, and 160 Hz, left to right) are shown. The red rectangles represent the corresponding median values.

Thus far, we have shown that our model neurons exhibit signature electrophysiological and encoding characteristics of CA1 place fields. Specifically, we showed that our place cell models exhibit electrophysiologically matched intrinsic properties (Figs. 5–7), also manifesting neuron-to-neuron heterogeneities in these intrinsic properties [36,37,56,57,67,68] through variable expression of ion channels in these neurons. Importantly, the kinetics and gating properties of these ion channels and the range of intrinsic heterogeneities were derived from CA1 pyramidal neuron electrophysiology. The incorporation of these intrinsic heterogeneities provided the substrate for us to demonstrate the need for strong synergy between intrinsic and synaptic properties to achieve efficient phase-coding models [Fig. 5(i)]. We showed that our models matched place field firing rates [Figs. 1(d), 5(a), 5(b), and 6] and experience-dependent asymmetry [31,33,65,66] in their firing rate profiles (Fig. 6). These place-cell electrophysiological characteristics that our models matched were in addition to the core focus of our study on phase precession [28,30,33], achieved by the emergence of the extracellular theta with a frequency that was *lesser* than the intracellular theta frequency [Figs. 1(f) and 1(g)]. In addition to these characteristics, place cells exhibit strong theta power and manifest a subthreshold ramp in their voltage response during place field traversals [33]. To further validate our models against these characteristics, we assessed theta power and subthreshold ramps in our model voltage responses and asked if they matched their electrophysiological counterparts. We performed these for each of the 132 models, with $F_{\mathrm{pre}}^{\max}$ set to three different values and with both symmetric as well as asymmetric synaptic activation profiles (Fig. 7).

We analyzed theta modulation and theta power by bandpass filtering the voltage response between 4 and 10 Hz. The subthreshold voltage response profile, to verify the presence of and quantify subthreshold ramps, was computed by median filtering the voltage response trace. The raw voltage responses, the theta-filtered traces, and the subthreshold response profiles for an example model neuron, receiving symmetric [Fig. 7(a)] and asymmetric [Fig. 7(b)] synaptic activation profiles for the three different values of $F_{\mathrm{pre}}^{\max}$ (40, 80, and 160 Hz) confirmed that our models manifested signature electrophysiological characteristics. Specifically, we observed that the theta-filtered traces showed a clear increase in theta power in the voltage response during place field traversals, with the spectra consistently showing peak power at 8 Hz. We also noted a consistent subthreshold response profile of 5–8 mV amplitude that was either symmetric [Fig. 7(a)] or exhibited an asymmetric ramp [Fig. 7(b)] depending on the nature of synaptic activation.

We noted that both the theta modulation (amplitude of the band-pass filtered signal) and the membrane potential ramp amplitude were comparable to those observed in place cells *in vivo* [33]. However, it may be noted that the out-of-field theta power in our model is zero, because our model assumes the out-of-field theta input to be zero, driven by our focus on spatial encoding *within* a single place field. The addition of spatially uniform inhibitory theta rhythm [54] to our formulation would ensure nonzero theta power outside the place field. However, the incorporation of such a spatially uniform inhibitory term to Eq. (6) would only contribute a small additive bias to the overall theta power computation. Therefore, we note that the *difference* in theta power within the place field, compared to outside, reported here is comparable to their experimental counterparts.

We quantified these measurements for all the 132 models that are both information efficient and intrinsically valid, for all the six cases (three different values of $F_{\mathrm{pre}}^{\max}$ for symmetric and asymmetric activation profiles) of synaptic activation profiles. We computed the area under the curve (AUC) of the Fourier spectrum of the theta-filtered voltage response [Fig. 7(c)], the ramp amplitude [Fig. 7(d)], and the asymmetry index [Eq. (20), Fig. 7(e)] from the correspondingly filtered versions of these voltage responses. Our results confirmed the expression of theta modulation and high theta power during place field traversals across all models [Fig. 7(c)]. We also confirmed that there was always a depolarizing subthreshold voltage profile during place field traversals that formed the substrate for action potential firing [Fig. 7(d)]. This subthreshold voltage profile was an asymmetric ramp [Fig. 7(b)] showing a strong asymmetry index [Eq. (20)] when the model was presented with asymmetric synaptic activation profiles [Fig. 7(e)]. Together, our model measurements matched their biological counterparts with reference to the expression of strong theta modulation and the manifestation of a depolarizing subthreshold ramp during place field traversals of an animal.

## VII Discussion

We built a conductance-based model for phase precession in CA1 pyramidal neurons, and derived a model-independent generalized quantitative measure for efficient phase coding within single place fields using information-theoretic approaches. We assessed the cellular and molecular constraints that are essential for obtaining highly efficient phase codes, employing a heterogeneous population of biophysically and physiologically constrained models. The prime conclusion of our study is that phase coding in place cells is critically reliant on a synergistic balance between neural gain and strength of external inputs, and that efficient information transfer through such a phase code could be achieved through multiple disparate routes while concomitantly maintaining signature excitability properties. These analyses unveil a role for neuronal intrinsic properties in maintaining a tight counterbalance to the overall afferent synaptic drive in achieving encoding efficiency. Importantly, this synergistic balance was found exclusively in models exhibiting efficient phase coding, and not for mere maintenance of excitability homeostasis. These conclusions on a role for neuronal intrinsic properties in regulating phase coding efficiency were possible because we had employed a population of models that incorporates biological heterogeneities in intrinsic excitability, rather than using a single hand-tuned neuronal model.

We arrived at these conclusions by employing an unbiased stochastic search across parameters involving thousands of models to ensure that we capture biological neuron-to-neuron heterogeneities. Our stochastic search involved an 11-dimensional parametric space, and demonstrated the absence of any strong constraints on ion channel expression in the emergence of efficient phase coding. Importantly, our analyses unveil an important cellular constraint involving a negative slope manifold in the two-dimensional intrinsic-synaptic functional plane in achieving efficient phase coding. This critical cellular constraint was achievable with disparate parametric combinations, thereby resulting in ion channel degeneracy that defined the emergence of efficient phase coding. Finally, by modifying the symmetry properties of the afferent inputs, we demonstrated that asymmetry in place field afferent inputs introduces predictive temporal shifts to the rate and phase codes. We noted that the shift in the phase code constitutes an adaptive shift to preserve phase-code efficiency in a manner that was driven by afferent stimulus statistics.

### A Synergistic interactions between intrinsic and synaptic properties drive phase coding efficiency

Our results make a clear case for phase precession and the efficiency of the associated phase code to be regulated by neuronal intrinsic properties, rather than being solely reliant on the temporal structure of the afferent network inputs. Employing models that received afferent inputs with identical temporal structure, we showed that neuronal intrinsic properties are critical in achieving efficient phase coding. Importantly, this dependence was not driven by a simple correlation between efficient phase coding and neuronal excitability (Figs. 5 and S3 [55]) or between phase-coding efficiency and firing rate during place field traversal [Fig. 5(a)]. Instead, phase-coding efficiency and associated parametric degeneracy were mediated by synergistic interactions between intrinsic and synaptic properties, specifically pointing towards the ability of intrinsic excitability and synaptic drive to *counterbalance* each other in achieving this [Fig. 5(i)]. Based on these observations, we postulate that the emergence of stable, efficient, and robust encoding in neuronal systems relies on synergistic interactions between disparate forms of plasticity. Under such a postulate, the specific forms of plasticity that define such emergence would be variable in a neuron-and context-dependent manner, depending on the internal state of the network (given parametric degeneracy) and on the afferent modulation imposed by behavior. Future studies could explore the manifestation of such counterbalances in intrinsic vs synaptic characteristics, and their roles in regulating encoding *and* homeostasis under physiological or pathophysiological conditions where these characteristics are known to undergo changes.

Our model presents a quantitative testable prediction on the dominant impact of SK channels on phase coding. Future computational studies could focus on whether this dominant role is a reflection of the slow kinetics of the SK current, or if this were just a reflection of the SK current altering neuronal excitability. Experimentally, electrophysiological recordings of phase precession within a single place field (in different cell types) could employ the generalized information metric derived here for assessing spatial information in and efficiency of the phase code. Such experiments could also test the role of different ionic currents (including SK) on phase coding in place cells with specific pharmacological agents or specific transgenic mice that have altered channel conductance or properties.

### B Degeneracy in efficient coding and excitability robustness

We show that the emergence of efficient phase coding (Fig. 2) and *concomitant* excitability robustness (Fig. 3) is independent of the ability of a neuron to maintain its ion channel densities at *specific* values but was driven by synergistic interactions between synaptic and intrinsic properties [Fig. 5(i)]. Such degeneracy implies that there are several degrees of freedom available to a neuron in concomitantly maintaining efficiency of the phase code *and* homeostasis of intrinsic excitability, without cross interferences between the encoding and the homeostasis processes. This constitutes an important departure from conventional analyses of the encoding-homeostasis balance, where encoding is hypothesized to be achieved by *specific* processes and *other* concurrent (or slower) processes achieve homeostasis. Within our framework, encoding and homeostasis is postulated to emerge concomitantly, with significant *degeneracy* in the specific components that contribute to such emergence. This degeneracy was effectuated by the ability of disparate combinations of *molecular constituents* to satisfy the *cellular-scale constraint* on counterbalancing afferent synaptic drive and matching excitability properties of CA1 pyramidal neurons.

Our analyses also constitute a scenario where redundancy reduction with reference to a code is brought about by degeneracy in the structural components that contribute to the emergence of the code. It is important to note that our analysis does not constitute coding degeneracy, where disparate codes (potentially mediated by different structural components) encode the same stimulus or structural redundancy, where a dysfunctional component is replaced by an identical component that restores function. Ours is an example of a scenario where an efficient code, that reduces redundant representations, is achieved by disparate combinations of underlying structural components (channels and receptors). Similar to other examples of degeneracy across the literature [34,35,58,59,62,63], while the contribution of different structural components to individual models is variable, the specific function that emerges as a consequence of interactions between these distinct structural components remains *precise and well defined*.

### C Models for phase precession

There are several competing models in the literature for how phase precession could emerge: the dual oscillator interference model [23,69], the modified dual oscillator model [70], the soma-dendritic interference model [71–74], and network [75–78], experience-dependent [31], and inheritance-based [79,80] models. There are ongoing debates about which of the several models best explain the experimental observations. Our model builds on an existing rate-based model [30] to formulate a conductance based model for phase precession that specifically accounts for several observations on place cell firing and electrophysiology: the emergence of phase precession from exact spike timings in conductance-based models with neuron-specific ion channels and receptors (Figs. 1 and 2); biological heterogeneities in neural excitability (Figs. 3 and 4); the emergence of subthreshold voltage ramp (Fig. 7), increased firing rate (Fig. 6), enhanced theta power within the place field (Fig. 7); a signature reduction in extracellular theta frequency compared to its intracellular counterpart [Figs. 1(f) and 1(g)]; and experience-dependent asymmetry in firing rate profile (Figs. 6 and 7).

Intuitively, the higher frequency of the intracellular theta (compared to the extracellular theta, which acts as the reference oscillation for assessing phase precession), in conjunction with the ramp in the voltage driven by afferent synaptic inputs, explain phase precession in this model [30,33]. Specifically, the extracellular theta oscillations are *slower* [*f*_θ_= 7.57 Hz; Figs. 1(f) and 1(g)], owing to the well-defined temporal structure [Eqs. (12) and (19)] of inputs from the presynaptic CA3 neurons. The intracellular theta, which is *slightly faster* (in our case *f*_0_ = 8 Hz), drives spiking close to the *peaks* of the intracellular oscillation. In addition, the depolarization driven by afferent synaptic drive enhances theta power during place field traversal, thereby increasing spike probability and allowing spikes to occur earlier than the peak of the extracellular theta. Therefore, with reference to the slower extracellular oscillation, these spikes show precession in phase space. Thus, in our model, the input structure from CA3 which accounts for the *T — τ* relationship and the depolarization ramp [Gaussian in Eq. (12) and Erlang in Eq. (19)] *mediates* phase precession. Our systematic stochastic search demonstrated the requirement of a synergistic balance between intrinsic excitability and synaptic strength in preserving the ability of this input structure in mediating phase precession and in yielding efficient phase coding through disparate routes.

Do our conclusions on phase coding depend on the specific choice of the model for phase precession? In the absence of heterogeneous conductance-based implementations of each of these different models, it is infeasible to make predictions on whether our conclusions would depend on the specific model in hand. However, we postulate that our main conclusions should hold irrespective of model choice. First, we postulate the requirement of a balance between synaptic drive and intrinsic excitability as a general principle for achieving efficient phase coding. Second, we hypothesize that ion-channel degeneracy would play a key role in the emergence of concomitant efficient phase codes and signature electrophysiological properties. We base this hypothesis on the several instances across different systems demonstrating ion-channel degeneracy in achieving cellular-and network-scale functions [35,60–62,81–85]. Further theoretical explorations could implement the different models for phase precession within a heterogeneous conductance-based setting to directly test these postulates and to assess the role of specific ion channels on phase coding efficiency.

### D Excitation-inhibition-intrinsic excitability (E-I-IE) balance

There are several competing models on the specific roles of the balance between excitatory and inhibitory synaptic inputs in the emergence of place cells and phase precession [30,31,54,70,71,73,79,80,86–92]. Although our model doesn’t explicitly account for inhibition, the parameter *P*_max_ [Eqs. (8)–(10)] could be interpreted as the strength of the net synaptic drive. This is feasible because there are lines of evidence that inhibition is spatially uniform [54], and the addition of uniform rhythmic inhibition to our model equation [Eq. (6)] would only serve as a bias term within the place field, and could be absorbed into the leak term within the framework.

An important methodological variation in our study is the systematic incorporation of biological heterogeneities in ion channel conductances and in neuronal intrinsic properties. This is in contrast to models for phase procession, which employ a single hand-tuned model with fixed channel conductances and intrinsic excitability. This methodological variation allowed us to demonstrate a *modulatory* role for intrinsic excitability in the emergence of phase precession, while also ruling out a direct well-defined relationship between intrinsic excitability and phase-coding efficiency [Figs. 5(c)–5(e)]. Importantly, incorporation of heterogeneities in intrinsic excitability and in synaptic strength allowed us to demonstrate the *synergistic counterbalance* between intrinsic excitability and synaptic strength for the emergence of efficient phase coding systems. Explicitly, as in [30], the temporal structure of CA3 inputs [defined by Eqs. (12) and (19)] *mediates* phase precession in our model. Here, we demonstrate that neuronal intrinsic excitability and synaptic strength play *modulatory* roles in preserving the ability of this input structure in mediating phase precession and unveil the requirement for a synergistic counterbalance between them in maintaining high efficiency phase coding. These observations emphasize the need to broaden the scope of the definition of excitation-inhibition (E-I) balance to include neuronal intrinsic excitability (IE) in the assessment of phase coding in particular, and in neuronal and network physiology and pathophysiology in general.

The discoveries of physiological roles for active dendritic structures in information processing [14,93–98], of behavior-driven activity-dependent plasticity in ion channels and intrinsic excitability [36,37,99–103], and of channelopathies that are associated with several neurological conditions [104–110], have emphasized the need to broaden the scope of a *passive* balance between excitatory and inhibitory synaptic inputs. Neuronal intrinsic properties—including neural excitability, frequency-dependent filtering, spatiotemporal summation, dendritic spike, and plateau potential initiation— could dramatically alter neural function even when identical inputs arrive onto neural structures. Therefore, studies assessing neural coding should assess an *active* balance that incorporates neuronal intrinsic excitability and ion channel function as a part of this balance. These studies should incorporate the tremendous biological heterogeneities observed in neural excitability, synaptic strength, and ion channel properties, rather than assuming a single model with fixed excitability and/or fixed excitatory/inhibitory synaptic strengths. Together, our study strongly suggests that a *passive E-I balance* be instead replaced by an *active E-I-IE balance* in the assessment of neural and network structures under physiological as well as pathophysiological conditions.

### E Limitations of our model and future directions

While emphasizing the phase code from a single neuron perspective, our approach did not account for the rate code or for phase and rate codes from a network perspective where different neurons together encode space by representing different place fields in an arena [31,69,111]. The analyses of encoding efficiency that accounts for multineuronal rate and phase coding is an important step forward. Such analyses should assess the specific roles of neuronal intrinsic properties in the concomitant emergence of efficient rate and phase codes across neurons, along with efficacious maintenance of intrinsic neuronal excitability across the network. These analyses would also provide avenues for assessment of degeneracy in a network-coding framework, for analyzing the role of heterogeneities in place field properties (e.g., in peak firing rates of individual neurons, extent of individual place fields, and the slope of phase precession), and for the study of potential relationships between phase-coding efficiency at a single-neuron scale and network scale, spanning a large spatial arena. From a broader perspective, our analyses here focus only on molecular and cellular constraints in achieving efficient phase codes of external space. However, the hippocampal formation has been implicated in other functions, such as recognition, completion, and separation of patterns and in engram formation. Future studies should therefore focus on the possibility that there could be other molecular and cellular constraints that define the hippocampal architecture towards satisfying these additional functions as well.

In the context of experience-dependent asymmetry, our model predicts a temporal adaptation that preserves the efficiency of the phase code when the symmetry of afferent synaptic drive is altered (Fig. 6). Our results show an expected predictive temporal shift in the rate and phase codes, with a stimulus statistics-dependent adaptation that preserved phase-coding efficiency (Fig. 6). However, it is important to note that the asymmetric afferent drive is just one of the physiological attributes that change with experience, with other attributes such as the somatodendritic inhibitory tone [91], the overall afferent drive, and dendritically initiated spiking [92] also exhibiting changes with experience. Although we report stimulus-dependent adaptation in the phase code that preserved its efficiency with the asymmetry, experience dependence might alter or preserve the efficiency of the phase code through any of these other experience-dependent changes, which have not been incorporated into our model. Therefore, future models could assess experience dependence of phase coding efficiency (and potential mechanisms that preserve efficiency) by accounting for all aspects of experience dependence, rather than assessing only asymmetric afferent drives [31,65,66,91,92].

Accounting for these aspects of experience dependence also requires that the place-cell model accounts for morphological properties of CA1 pyramidal neurons [63], including localization and activation profiles of intrinsic [57,63,112] and synaptic [54,63,113–116] properties. Incorporation of these components into morphologically realistic neurons will enable the computation of extracellular theta waveforms using forward modeling approaches for local field potentials [116,117], rather than using population firing as a proxy for the extracellular theta. Such models, apart from exploring the roles of E-I-IE balance and degeneracy in efficient phase coding, could also assess potential roles for intrinsic neuronal properties (and specific ion channels that mediate them) in amplifying and extending the range of phase precession (similar to the role for spatially uniform inhibition demonstrated in [54]). We postulate that the degeneracy in efficient phase coding and concomitant robustness in intrinsic excitability, emergent due to synergistic interactions between neural gain and external inputs, would span more structural components and express more effectively if these additional components and associated interactions were introduced into our model.

Our model was limited to phase precession in CA1 pyramidal neurons, focusing on heterogeneities in intrinsic properties owing to baseline variability, activity-dependent plasticity, or neuromodulation. We postulate that our fundamental conclusions would extend to other neuronal subtypes that exhibit phase precession. Specifically, we hypothesize that synergistic interactions between neural gain and external inputs would mediate the expression of degeneracy in the concomitant emergence of efficient phase coding and homeostasis in other neuronal subtypes as well. Such degeneracy would be achieved through disparate combinations of *specific* structural components expressed in those neuronal subtypes, and should be explored through careful assessment of intrinsic-synaptic counterbalances mediated by ion channels and receptors expressed in those neurons. However, such generalization should specifically account for differences in local/afferent connectivity (including recurrent connections) as well as the specific intrinsic properties and ion channels expressed in these neurons.

## Supplementary Material

Supplemental Figures S1–S5

## Figures and Tables

**Table I:** Parameters and their ranges governing the multiparametric multiobjective stochastic search.

No.	Parameter	Minimum value	Maximum value
1	Sodium conductance, gNa (mS/cm^2^)	1	100
2	Delayed rectifier potassium conductance, gKDR (mS/cm^2^)	0.1	100
3	*A* type K^+^ conductance, gKA (mS /cm^2^)	0	10
4	*L* type Ca^2+^ conductance, gCal (*μ*S/cm^2^)	0	100
5	Calcium gated K+ conductance, gSk (*μ*S/cm^2^)	0	100
6	HCN conductance, gh (*μ*S/cm^2^)	0	100
7	*M* type K^+^ conductance, gKM (*μ*S/cm^2^)	0	100
8	*T* type Ca^2+^ conductance, gCaT (*μ*S/cm^2^)	0	100
9	Calcium decay constant, *τ*_Ca_(ms)	20	100
10	Specific membrane resistance, *R*_m_(kΩ cm^2^)	20	60
11	Synaptic permeability, *P*_max_(nm/s)	0.2	2

**Table II:** Electrophysiological bounds on intrinsic measurements employed for model validation.

No.	Intrinsic measurement	Lower bound		Upper bound
1	Mean resting membrane potential, *μ*_RMP_(mV)	-75		-60
2	Standard deviation of resting membrane potential, σ_RMP_(mV)		<1	
3	Input resistance, *R*_in_ (MΩ)	30		120
4	Firing rate at 50 pA, *f*_50_(Hz)		=0	
5	Firing rate at 250 pA, *f*_250_ (Hz)	1		45
