# Supplementary material for: Efficient phase coding in hippocampal place cells
Source: Phys Rev Res. Author manuscript; Available in PMC 2020 Sep 24. (PMC7116119; doi:10.1103/PhysRevResearch.2.033393)
Supplement: Supplemental Figures S1–S5 [file EMS94969-supplement-Supplemental_Figures_S1_S5.pdf]

Efficient phase coding in hippocampal place cells

Pavithraa Seenivasan and Rishikesh Narayanan

Cellular Neurophysiology Laboratory, Molecular Biophysics Unit, Indian Institute of Science, Bangalore 560012, India.

Supplemental Material

Supplemental Figure S1 2
Supplemental Figure S2 3
Supplemental Figure S3 4
Supplemental Figure S4 5
Supplemental Figure S5 6

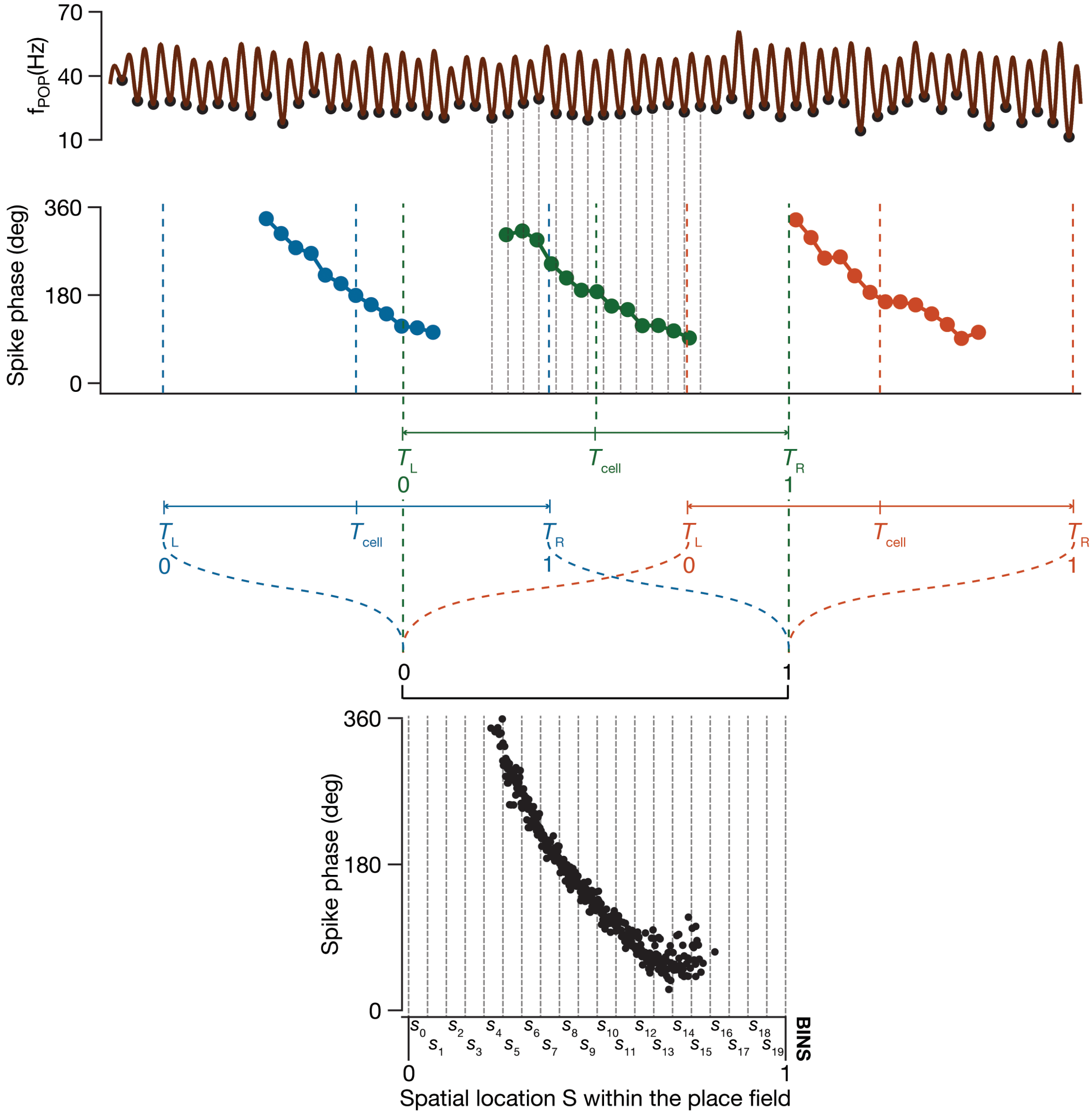

Supplemental Figure S1. Illustration of computing the phase-space plot within our modeling framework. The population firing frequency (f_{POP}) constituted the reference theta (first row, brown), which was computed from the spikes corresponding to all 50 spatially-distinct place-field traversals. Spike phases (second row; blue, green and orange dots denote spike phases for three representative traversals) corresponding to each place field traversal were computed with reference to the detected troughs (top, black circles) of f_{POP}. Each place-field traversal had a place field center (T_{cell}), a left extreme (T_L) and a right extreme (T_R) beyond which there can be no spike corresponding to that traversal. T_{cell}, T_L and T_R for each of the three representative place-field traversals are demarcated with color-matched dotted lines. The T_L to T_R extremes of each place field traversal are then mapped respectively to 0 and 1 on normalized spatial scale (S), with T_{cell} falling at 0.5 on this normalized scale. The spike phases corresponding to multiple traversals are then superimposed onto this normalized scale to yield the phase-space plot (third row). The spatial scale is split into 20 different bins (third row) for computing the probability distributions that are used to calculate the efficiency of spatial information transfer through this phase code.

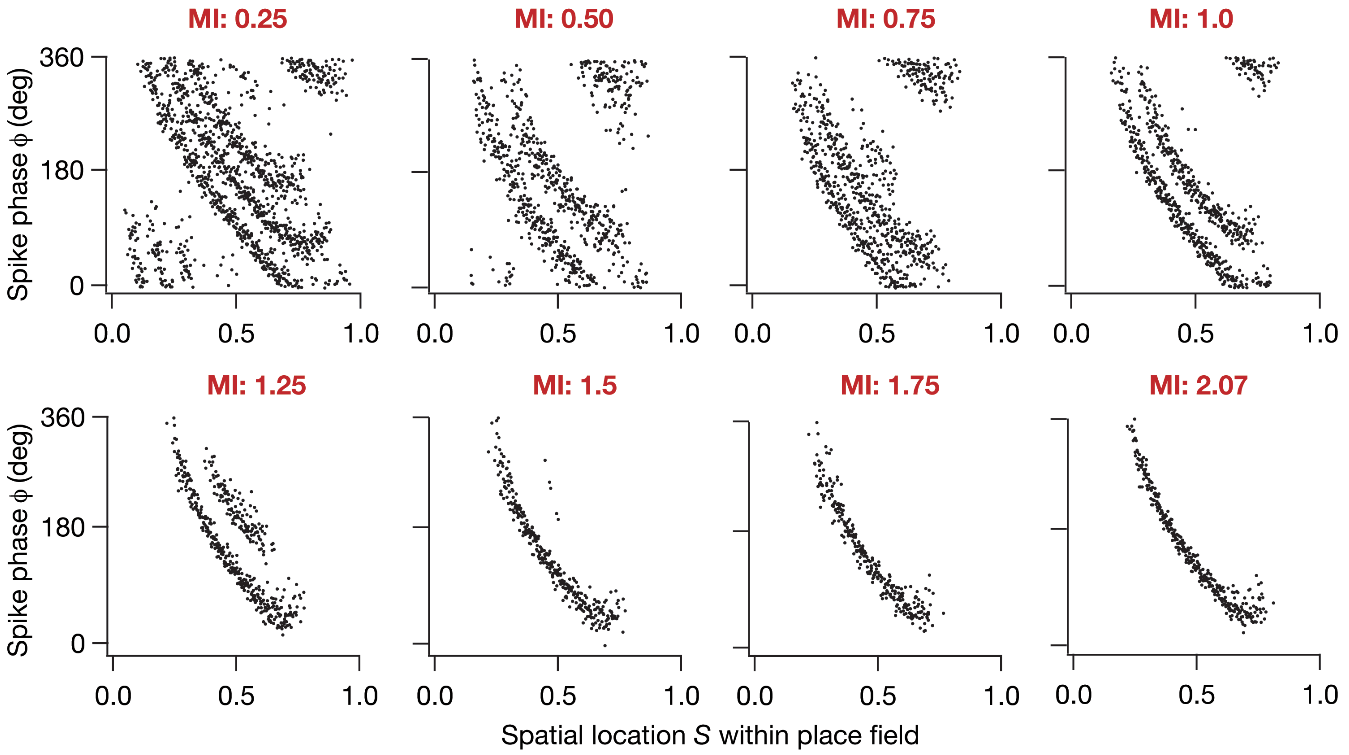

Supplemental Figure S2. Spatial information transfer through spike phases was differential across models obtained from the unbiased stochastic search. Phase precession of eight example model neurons with successively higher mutual information values, picked from the pool of 11,000 models that were generated as part of the stochastic search procedure. The threshold for choosing efficient models was set at 1.5 bits of MI, implying that only the last three of the models above will be chosen as efficient models.

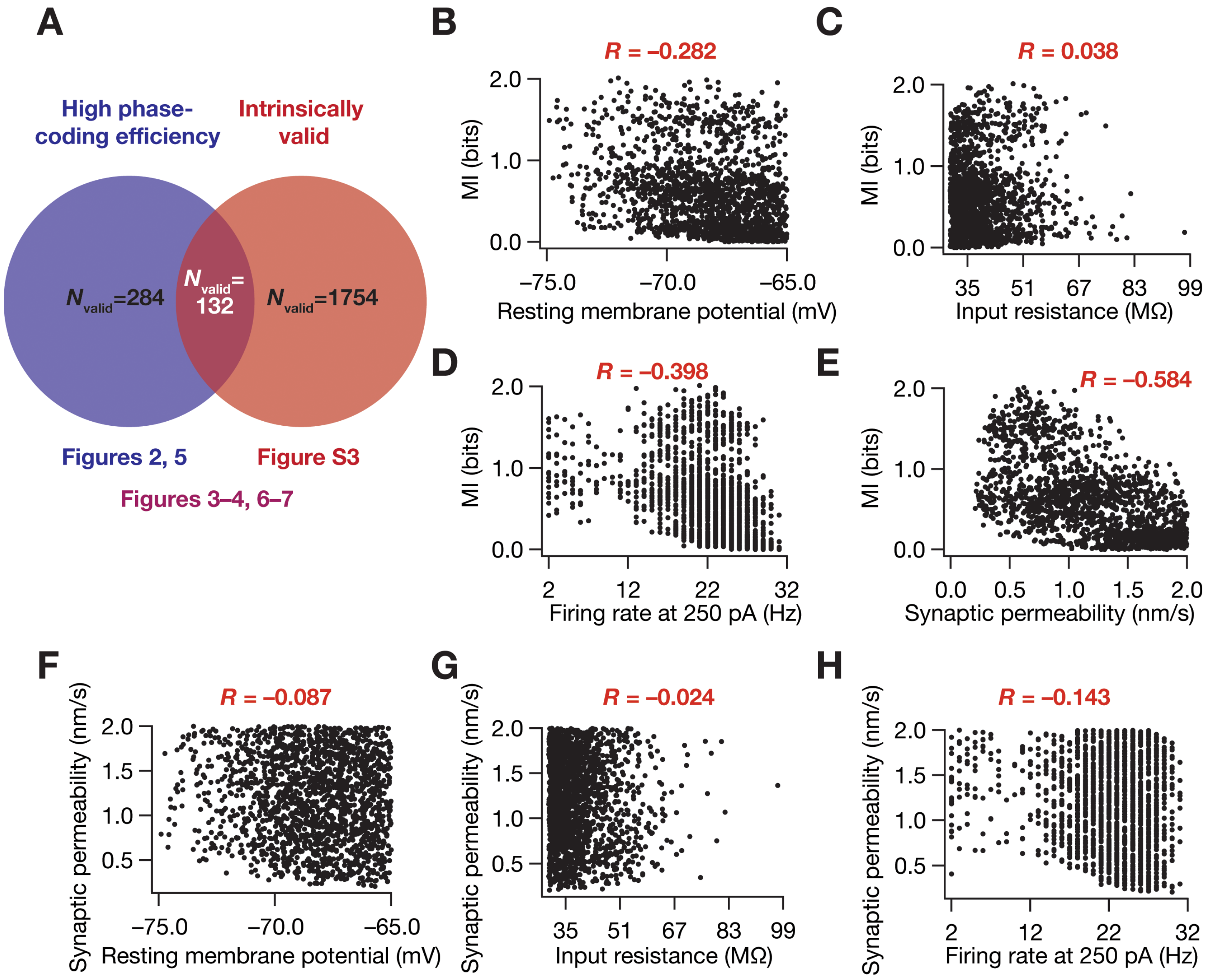

Supplemental Figure S3. Efficiency in phase coding was not strongly correlated with intrinsic excitability or synaptic strength in models with signature excitability characteristics of CA1 pyramidal neurons. (A) For other analyses in the study, as a first step, we had found efficient models with high MI (N_{valid}=284; blue circle) from the 11,000 stochastically generated models (Fig. 2). As a second step in such analyses, we found models with concomitant signature excitability characteristics as a *subset* (N_{valid}=132; purple intersection region between the red and blue circles) of these high-MI efficient models (Fig. 3). For the analyses presented in this figure, instead of finding efficient models as the first step, we found models among the entire set of 11,000 stochastically generated models that satisfied signature excitability characteristics. This validation procedure yielded 1754 models that were intrinsically valid (red circle), but did not have any constraints imposed on their efficiency (mutual information). The analyses presented in this figure correspond to these 1754 intrinsically valid models. (B–E) Scatter plots of mutual information *vs*. resting membrane potential (B), input resistance (C), firing rate at 250 pA (D) and synaptic permeability (E) unveiled the absence of strong correlations between efficient coding and intrinsic/synaptic functional properties. (F–H) Scatter plots of synaptic permeability *vs*. resting membrane potential (F), input resistance (G) and firing rate at 250 pA (H) revealed the absence of strong correlations between intrinsic properties and synaptic strength. The values against *R* represent Pearson's correlation coefficients for the respective color-coded scatter plots. N_{valid}=1,754 for all plots.

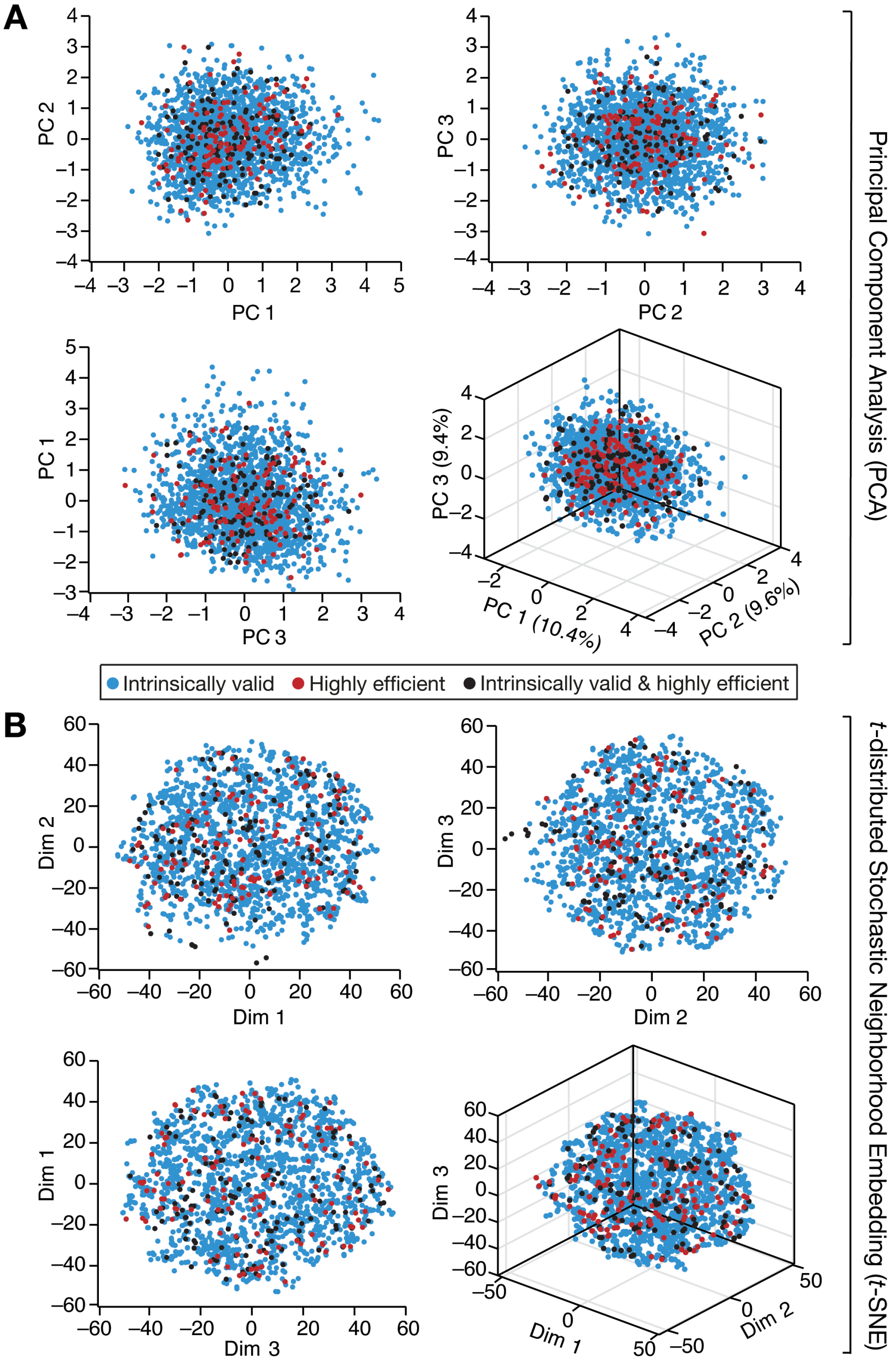

Supplemental Figure S4. Dimensionality reduction of the 11-dimensional parametric space unveiled overlapping parametric distributions across efficient and/or intrinsically valid models. Outcomes of principal component analysis (A) and *t*-distributed stochastic neighbor embedding (B) on the eleven-dimensional parametric space (Table 1) of 8579 models that yielded place-field driven spikes. The coefficients on the first three principal dimensions, associated with three subsets of models, are represented with filled circles of three colors: blue represents models that are intrinsically valid but not efficient phase coders (N_{valid}=1622), red represents models that are efficient phase coders but not intrinsically valid (N_{valid}=152) and black represents models that are both efficient and intrinsically valid (N_{valid}=132). Two-dimensional plots of the three principal dimensions (A, B: top–left, top–right, and bottom–left) and a three-dimensional plot showing all the three principal dimensions (A, B: bottom–right) of the same data, obtained with PCA (A) or *t*-SNE (B) revealed the lack of clustering corresponding to the three groups of models. Coefficients corresponding to models that were neither efficient nor intrinsically valid (N=6673) also overlapped with these three populations (not shown). These analyses unveil a lack of a defined structure in the parametric space that distinguishes these model subsets.

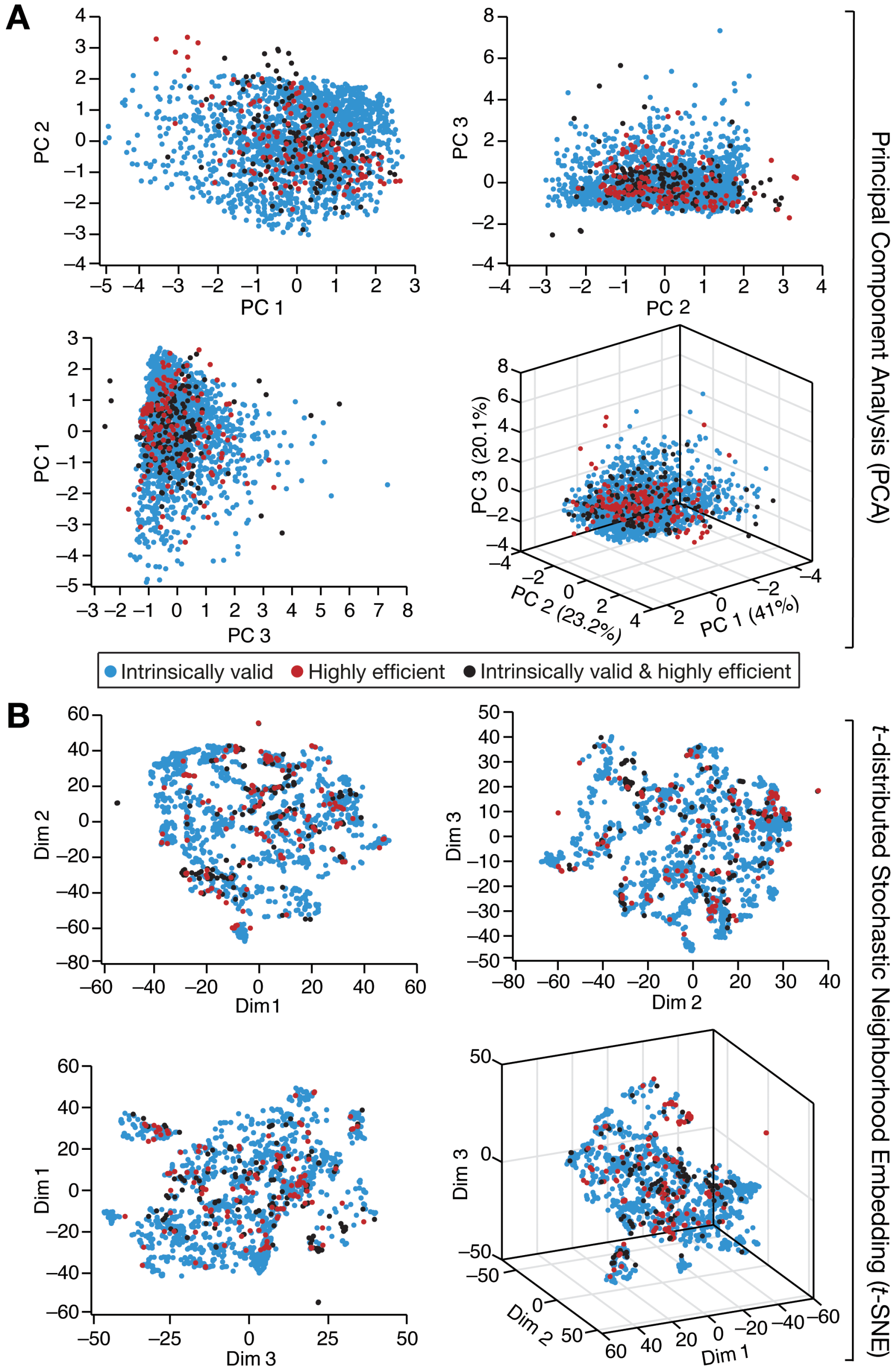

Supplemental Figure S5. Dimensionality reduction of the 5-dimensional measurement space unveiled overlapping distributions of measurements across efficient and/or intrinsically valid models. Outcomes of principal component analysis (A) and *t*-distributed stochastic neighbor embedding (B) on the five-dimensional measurements space (RMP, R_{in}, f_{250}, P_{max} and MI) of 8579 models that yielded place-field driven spikes. The coefficients on the first three principal dimensions, associated with three subsets of models, are represented with filled circles of three colors: blue represents models that are intrinsically valid but not efficient phase coders (N_{valid}=1622), red represents models that are efficient phase coders but not intrinsically valid (N_{valid}=152) and black represents models that are both efficient and intrinsically valid (N_{valid}=132). Two-dimensional plots of the three principal dimensions (A, B: top–left, top–right, and bottom–left) and a three-dimensional plot showing all the three principal dimensions (A, B: bottom–right) of the same data, obtained with PCA (A) or *t*-SNE (B) revealed the lack of clustering corresponding to the three groups of models. Coefficients corresponding to models that were neither efficient nor intrinsically valid (N=6673) also overlapped with these three populations (not shown). These analyses unveil a lack of a defined structure in the measurement space that distinguishes these model subsets.
